# The Himalayan uplift and evolution of aquatic biodiversity across Asia: Snowtrout (Cyprininae: *Schizothora*x) as a test case

**DOI:** 10.1371/journal.pone.0289736

**Published:** 2023-10-24

**Authors:** Binod Regmi, Marlis R. Douglas, Karma Wangchuk, Zachery D. Zbinden, David R. Edds, Singye Tshering, Michael E. Douglas

**Affiliations:** 1 Department of Biological Sciences, University of Arkansas, Fayetteville, Arkansas, United States of America; 2 National Research & Development Centre for Riverine and Lake Fisheries, Ministry of Agriculture & Forests, Royal Government of Bhutan, Thimphu, Bhutan; 3 Department of Biological Sciences, Emporia State University, Emporia, Kansas, United States of America; National Cheng Kung University, TAIWAN

## Abstract

Global biodiversity hotspots are often remote, tectonically active areas undergoing climatic fluctuations, such as the Himalaya Mountains and neighboring Qinghai-Tibetan Plateau (QTP). They provide biogeographic templates upon which endemic biodiversity can be mapped to infer diversification scenarios. Yet, this process can be somewhat opaque for the Himalaya, given substantial data gaps separating eastern and western regions. To help clarify, we evaluated phylogeographic and phylogenetic hypotheses for a widespread fish (Snowtrout: Cyprininae; *Schizothorax*) by sequencing 1,140 base pair of mtDNA cytochrome-b (*cytb*) from Central Himalaya samples (Nepal: N = 53; Bhutan: N = 19), augmented with 68 GenBank sequences (N = 60 *Schizothorax*/N = 8 outgroups). Genealogical relationships (N = 132) were analyzed via maximum likelihood (ML), Bayesian (BA), and haplotype network clustering, with clade divergence estimated via TimeTree. Snowtrout seemingly originated in Central Asia, dispersed across the QTP, then into Bhutan via southward-flowing tributaries of the east-flowing Yarlung-Tsangpo River (YLTR). Headwaters of five large Asian rivers provided dispersal corridors from Central into eastern/southeastern Asia. South of the Himalaya, the YLTR transitions into the Brahmaputra River, facilitating successive westward colonization of Himalayan drainages first in Bhutan, then Nepal, followed by far-western drainages subsequently captured by the (now) westward-flowing Indus River. Two distinct Bhutanese phylogenetic groups were recovered: Bhutan-1 (with three subclades) seemingly represents southward dispersal from the QTP; Bhutan-2 apparently illustrates northward colonization from the Lower Brahmaputra. The close phylogenetic/phylogeographic relationships between the Indus River (Pakistan) and western tributaries of the Upper Ganges (India/Nepal) potentially implicate an historic, now disjunct connection. Greater species-divergences occurred across rather than within-basins, suggesting vicariance as a driver. The Himalaya is a component of the Earth’s largest glacial reservoir (i.e., the “third-pole”) separate from the Arctic/Antarctic. Its unique aquatic biodiversity must be defined and conserved through broad, trans-national collaborations. Our study provides an initial baseline for this process.

## Introduction

The Himalaya is the most extensive and recently evolved mountain system on Earth (length = 2,400km; width = 240km; elevation = 75–8,800m), with global significance underscored by large-scale lithospheric, cryospheric, and atmospheric interactions [1]. These have not only driven global climate, but also defined the cultural and biological endemism of the region [2]. The Himalaya is topographically separated into three components: Western Himalaya: ~880 km wide region framed by Indus and Kali Gandaki rivers (southwestern Tibet Autonomous Region of China to western Nepal, respectively); Central Himalaya: ~800 km wide area contained between the Kali Gandaki and Tista rivers (from western Nepal to Sikkim, India, respectively) and; Eastern Himalaya: ~720 km stretch delimited by the Tista and Brahmaputra rivers (from Sikkim to Arunachal Pradesh in India) (https://www.pmfias.com/himalayas-regional-divisions-punjab-himalayas-assam-himalayas-western-himalayas-central-himalayas-eastern-himalayas/). Massive, tectonically derived mountain chains such as the Himalaya are fundamental in establishing global biodiversity gradients, but strong signals of vicariance and local adaptation are generally noted within terrestrial rather than aquatic systems [3]. Here we evaluate how orogeny (the deformation and folding of Earth’s crust by lateral compression) has contributed to the potential diversification of freshwater fishes broadly across Asia by quantifying phylogeographic and phylogenetic patterns withing a an endemic high-elevation fish genus (*Schizothorax*). We use these data to infer a potential evolutionary hypothesis for the genus, and offer the resulting perspective as a surrogate for other aquatic biodiversity components in the region.

### Orogeny and biodiversity of the study region

**Fig 1:**
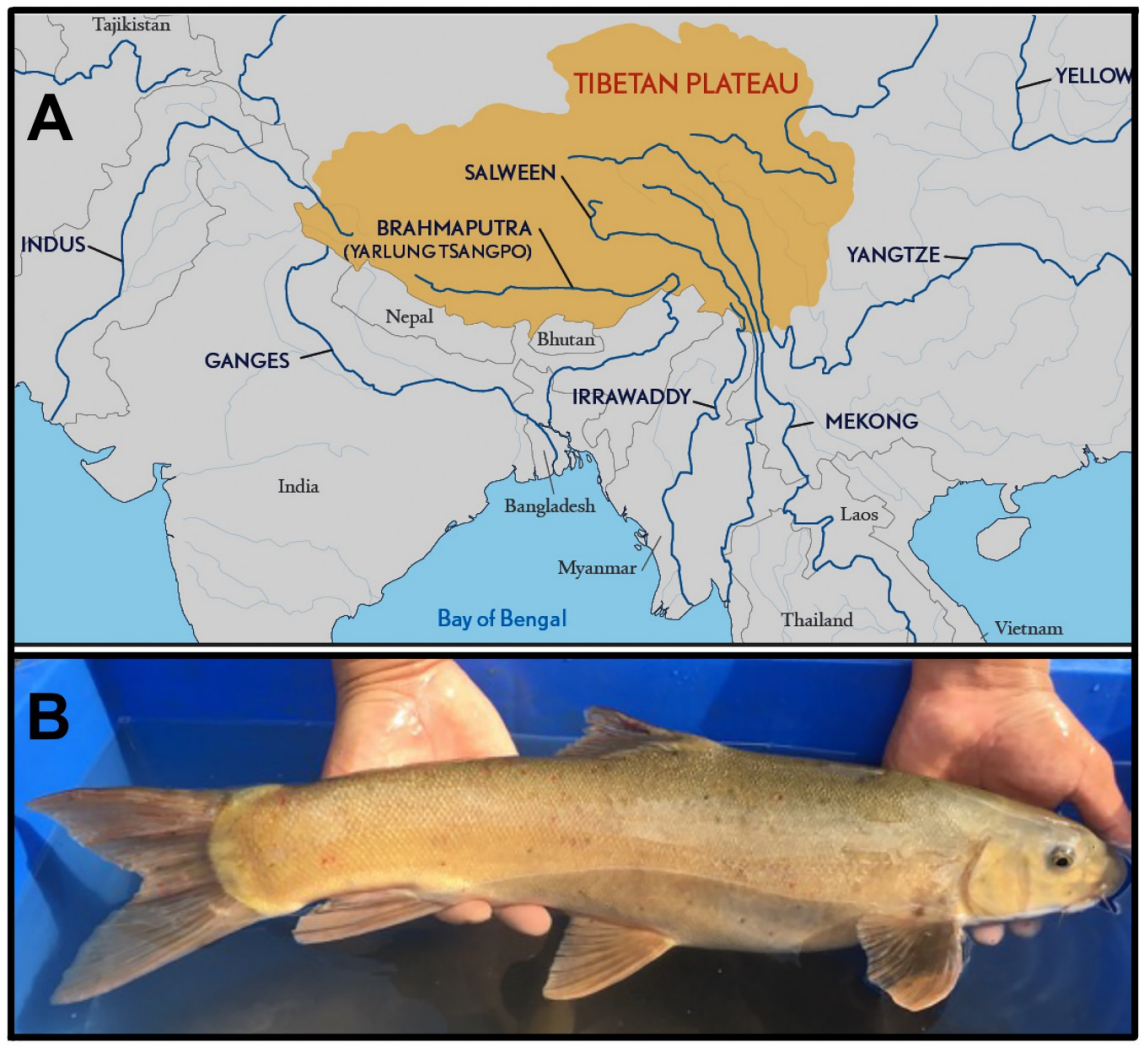
**(A) Simplified drainage map of Asia, showing major basins from which *Schizothorax* were obtained**. Area in yellow indicates the Tibetan Plateau, referred to as Qinghai-Tibetan Plateau (QTP) in China. The Tarim River, an endorheic basin north of the QTP in Central Asia, is not depicted. Three major rivers, the Irrawaddy, Salween, and Mekong, drain the QTP into Southeastern Asia (SEA), whereas the Yangtze River flows east into the China Sea (Republished from Fig 1 [100] under a CC BY license, with permission of the *Council on Foreign Relations*, original copyright 2016). **(B) *Schizothorax sp*. *cf richardsonii* (i.e., potentially *S*. *richardsonii*)** captured 02 December 2016 at confluence of Bibigang Chhu and Mangde Chuu, Bhutan (Total length = 540 mm).

The Himalaya formed as a series of parallel ranges bordered by rivers to the west (Indus) and east (Brahmaputra). It encompasses most of northern Pakistan, northern India (Kashmir to the west, Assam to the east), as well as Nepal, Bhutan, and parts of China, with the extensive Qinghai-Tibetan Plateau (QTP) to the north and the alluvial plains of India and Bangladesh to the south (Fig 1A). It evolved over a 30 Ma span, as one component of the extensive impact between the Indian craton and the Eurasian landmass [4].

This uplift established the Himalaya as a topographic entity and profoundly influenced the connectivity of its regional rivers [5, 6]. In late Oligocene–early Miocene (26–19 Ma), the Yarlung-Tsangpo River (= YLTR; average elevation 4,000m) formed on the southern border of the QTP as an east-west series of lakes with south-flowing tributaries (Fig 11 in [7]). These lakes eventually coalesced into a westward flowing river (early–mid Miocene; 19–15 Ma) that eventually reversed its flow in response to a continuing plateau uplift. The YLTR was captured on the eastern QTP by the Brahmaputra River in early-mid Miocene (i.e., ~18–15 Ma) [6, 8]. The uplift of the QTP was a signature event that seemingly allowed aquatic biodiversity to disperse broadly from west to east, with subsequent access to south-flowing Himalayan drainages eventually truncated by continued uplift [9].

Three stratigraphic zones subsequently emerged, as manifested by the contemporary species richness of terrestrial biodiversity [4]. Although this endemism has been hypothesized as driven by tectonic activity, requisite data are either sporadic or absent [10]. Freshwater fish divergence seemingly occurred concomitantly, with tributaries increasingly isolated as the QTP elevated more than 3,000m in late-Miocene [11]. The uplift also drove strong selection for high-elevation specialists that could subsist under more severe conditions [12]. However, data remain sparse for high elevation Himalayan fishes, an aspect inherently magnified by the lack of large-scale monitoring programs [13]. Such data-deficiencies, tightly linked with the geomorphic and climatic history of the region, underscore the strong potential for cryptic species yet to be identified and formally described.

### *Schizothorax* and its outgroups

Snowtrout (Fig 1B) offers a unique opportunity to deciphering the geologic processes that drove fish diversification and distribution in the Himalaya. The genus belongs to the largest and most diverse freshwater fish family (Cyprinidae: 1,700+ valid species, with 11 recognized subfamilies) [14], and comprises 28 recognized species across lakes and rivers of the QTP, the endorheic Tarim River basin, as well as the snow-fed tributaries of the Himalaya/ sub-Himalaya (Fig 1A) [15–17].

Several hypotheses have been offered to explain its origin and dispersal. Researchers first suggested the Schizothoracinae initially dispersed into the QTP from southeastern Asia [18], and subsequently became isolated by orogeny and climate change. Others proposed that a primitive barbin clade was isolated by tectonism on the QTP [19], thus serving as the progenitor for contemporary *Schizothorax*.

The subfamily Cyprininae was recently revised [20], based on a combination of molecular markers [i.e., *cyt-b* (N = 791); mitogenomes (N = 85); nuclear RAG-1 sequences (N = 97)], with *Schizothorax* being retained within the monophyletic tribe Schizothoracini, which evolved either pre- or post-Barbini, depending upon the molecular marker employed.

Six major biogeographic groups of Snowtrout have been proposed [21]: A Central Asiatic clade, followed by five drainage-specific sister-clades: YLTR/ Brahmaputra; Indus; Irrawaddy; Mekong/Salween; and Yangtze. However, this perspective remains topographically incomplete as data for major Himalayan drainages were unavailable. We extended these results by focusing on two previously unrepresented regions: Bhutan (eastern Himalaya) and Nepal (central Himalaya). In tandem, they link the far eastern Himalaya with its western terminus, thus allowing for a more complete evaluation of tectonism, orogeny, and the potential diversification of aquatic biodiversity in the region. By filling these biogeographic gaps, Snowtrout can be more appropriately recognize as a biodiversity component extending from Central Asia, across the QTP into eastern, southern, and western Asia. This, in turn, provides a formal template of regional biodiversity from which potential impacts of Quaternary climate oscillations can be more appropriately interpreted [22].

### Contemporary significance

The QTP (and surrounding mountains) are termed the “third pole” because the region contains the greatest amount of global snow and ice beyond the Arctic/Antarctic. It also represents the headwater source for 10 large Asian rivers (Fig 1A), which collectively provide ecosystem services for >1.5 billion people [23]. Glaciers in the eastern Himalaya, for example, represent an estimated 14.5% of the global total, a quarter of which has been lost since 1970 [24], due largely to a regional warming 2x greater than the global average [2]. These cumulative impacts are recorded as increasing precipitation, decreasing glacial surface area, diminishing snow cover, expanding glacial lakes, and a loss of permafrost [25].

These biophysical occurrences have substantially impacted the distribution of *Schizothorax*, provoking a migration into higher-elevation streams with a concomitant contraction of trailing-edge habitats [26]. This redistribution has also facilitated contact with invasive aquatic predators [27], and represents an essential element of ecological resilience for *Schizothorax* (e.g., its adaptive capacity). This shift further adds to the taxonomic uncertainties within the genus, as well as emphasizing the strong potential for unrecognized, cryptic diversity.

A comprehensive understanding of endemic biodiversity is a necessary requirement for holistic, scalable approaches that mitigate the impacts of regional climate-change. These must be derived from standardized metrics that quantify interspecific variation across political boundaries. Such an approach can provide templates for cooperative regional conservation [25] and help define the ecological resilience of native biota, against which climate impacts are defined and incorporated. Herein, our approach is to evaluate a biologically-complex genus of freshwater fish as a focal point for the development of such components.

## Materials and methods

### Ethics statement

All methods were performed in accordance with relevant guidelines and regulations. Nepali samples were collected as one component of a Fulbright Award to DRE (see Acknowledgments), whereas Bhutanese collecting permits were in conjunction with the National Research and Development Centre for Riverine & Lake Fisheries (NRDCR&LF), Ministry of Agriculture & Forests (MoAF), Royal Government of Bhutan. The export of fin clips was authorized through a Material Transfer Agreement (MTA) provided by the Bhutan Agricultural and Food Regulatory Authority (BAFRA). Sampling protocols were approved by the University of Arkansas Institutional Animal Care and Use Committee (UA_IACUC_17064).

Specifically, sampling was accomplished using standard fisheries methods, to include hand-held seines, dip nets, backpack shockers, as well as passive gear such as hoop/ trammel nets and minnow traps. These methods and devices are approved for use by the American Society of Ichthyologists and Herpetologists (ASIH; https://www.asih.org/resources) and the American Fisheries Society (AFS; https://fisheries.org/books-journals/online-resources/). In addition, educational resources [Association for the Advancement of Laboratory Animal Science (AALAS): https://www.aalas.org/] were employed to assist in the instruction of Bhutanese cadre.

Small-bodied fishes (<10 cm total length) were removed from capture devices with <20 placed concurrently within a 19-liter bucket of well-aerated stream water. Direct handling of fish was minimized, and individuals were retrieved via dip net for identification and subsequent release. Larger fishes were gently removed from capture devices and identified/ released at site of capture. Care was taken in all cases to ensure that water temperatures were identical between capture site and holding bucket. Once captured, fishes were continuously monitored, and euthanasia immediately employed if sampling injuries were detected. Fishes were so recognized if they could not maintain equilibrium in the holding bucket, and/or displayed erratic or impaired opercular movements (<20 respiratory cycles/minute). Euthanasia was conducted via an overdose of clove oil (Eugenol), followed by fixation in 10% formalin.

Clove oil satisfies specified criteria and is considered acceptable when used at high concentrations (>400 mg/L), as it causes a reliable and rapid loss of consciousness and induces hypoxia (critical components for eliminating potential pain; American Veterinary Medical Association Guidelines on Euthanasia; https://www.avma.org/sites/default/files/2020-02/Guidelines-on-Euthanasia-2020.pdf). A concentration of 50–150 mg/L was initially employed, then gradually increased to >400 mg/L through periodical additions (~50 mg/L). Complete cessation of opercular movement was used as an indicator of death, with the fish then being retained in solution for >10 additional min.

Tissues for DNA analysis were non-lethally sampled by removing a small piece of anal fin (a non-propulsive appendage). The fish was first placed in a small bucket of stream water treated with clove oil, and subsequently removed via dipnet immediately following a loss of righting ability. A small piece of fin was removed with surgical scissors cleaned in 100% EtOH. The clip was transferred to a Whatman FTA^©^ card for preservation and storage, and the fish revived in a small bucket of untreated stream water until righting ability was regained, at which time it was released at the collecting site. Wounds caused by clipping of fins will normally heal satisfactorily without application of antibiotics or topical analgesics, which are not recommended for use in wild fishes (per AFS resources).

### Sample sites: Drainages in Nepal and Bhutan

**Fig 2:**
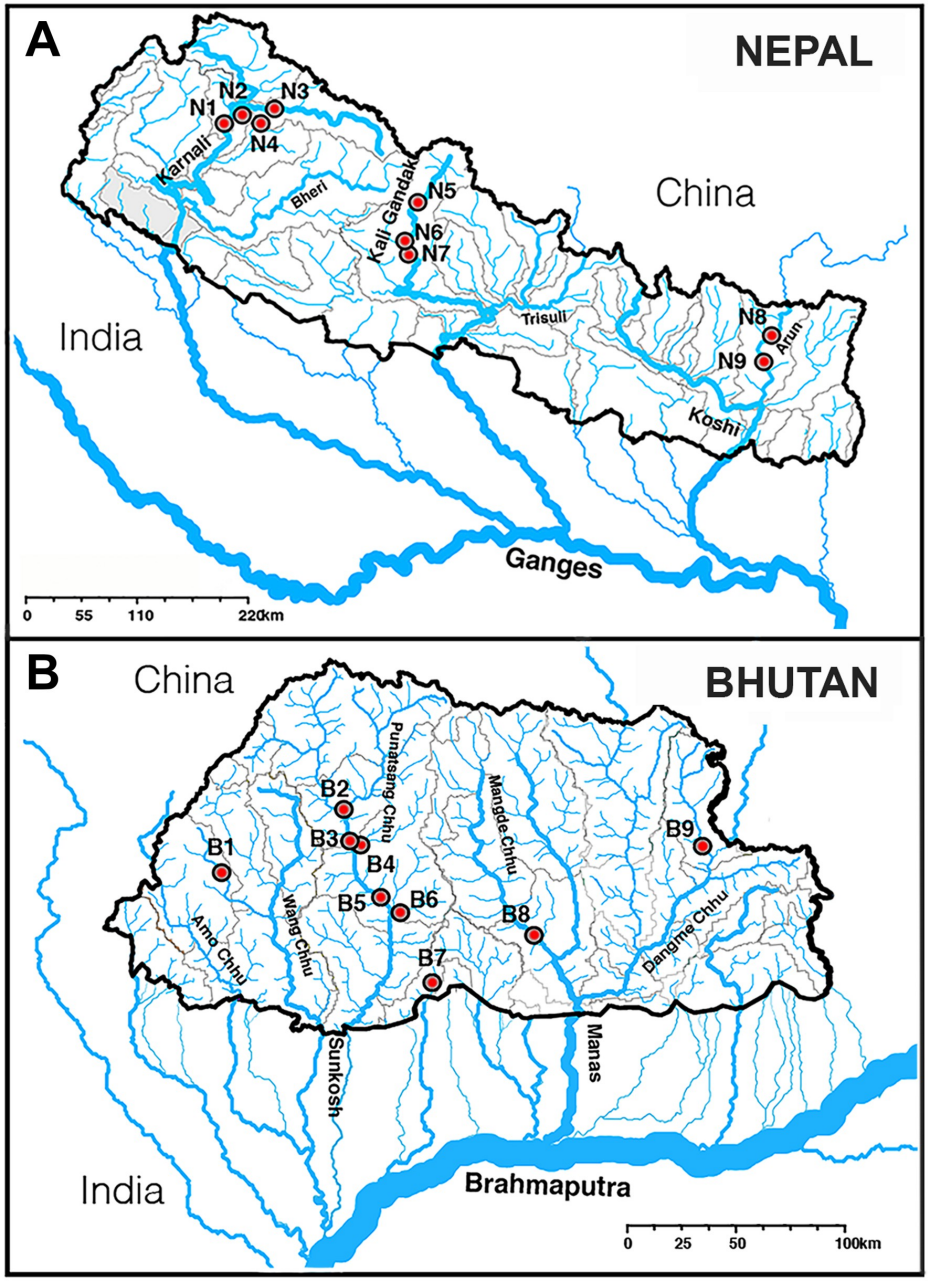
Map depicting sampling locations for *Schizothorax*, with metadata for ’Site,’ ’River,’ ’Location,’ ’Lat-Long,’ ’Haplotype,’ and ’Group’ compiled in S1–S3 Tables, respectively. Red circles in map indicate sampling sites with samples identified by Group number in (A) Nepali as N = 1–9, and (B) Bhutan as B = 1–9. Maps were generated in ArcGIS 10.4.1 using publicly available data from HydroRIVERS and HydroBASIN (https://www.hydrosheds.org/products/hydrorivers).

Nepal is drained by three major east-to-west rivers (Koshi, Gandaki, and Karnali; Fig 2A) that collectively support eight tributaries, several of which originate on the QTP. In eastern Nepal the stronger effects of the monsoon are reflected by more eroded and abruptly-elevated mountainous slopes of the Koshi River. Central Nepal, dominated by the Gandaki Basin and its western tributary (Kali Gandaki), serves as a distinct east-west vicariant break. Western Nepal is drained by the Karnali Basin and includes Rara Lake (2,990m elevation), a RAMSAR site with a surface area of 10.6 km^2^. All three basins drain south into the east-flowing Ganges River which joins with the Brahmaputra River to terminate in the Bay of Bengal (Figs 1 and 2A).

Bhutan has five major north-south river systems (Fig 2B): The Amo Chhu and Wang Chhu descend from the QTP, flow southeasterly through western Bhutan and then into West Bengal (India). The Punatsang Chhu (= Sunkosh) drains the Great Himalaya Range in west-central Bhutan, and flows into Assam (India). The Manas River and its major tributaries drain central-eastern Bhutan. The Dangme Chhu originates in Arunachal Pradesh (India) and flows southwesterly through Bhutan, whereas the Kuri Chhu (from the QTP), the Chamkhar Chhu, and the Mangde Chhu drain central Bhutan. The eastern-most river, the Nyera Ama Chhu, is the only drainage that originates entirely within Bhutan. All river systems eventually drain into the Brahmaputra River (northern India), which curves south and west through Bangladesh to merge first with the Ganges River before flowing into the Bay of Bengal (Figs 1, 2A and 2B).

### Amplification and sequencing of mtDNA samples

Nepali samples (N = 53; S1 Table, Fig 2A) represent five *Schizothorax* species. Three [*S*. *macrophthalmus* (N = 3), *S*. *nepalensis* (N = 4), and *S*. *raraensis* (N = 3); total N = 10] represent a landlocked species-flock within Rara Lake (Karnali River drainage; Fig 2A). Two other riverine species [*S*. *richardsonii* (N = 26), and *S*. *progastus* (N = 17)] were sampled from (west-to-east): Karnali River (N = 12/N = 3, respectively); Gandaki River (N = 11/9); Koshi River (N = 5/3). Nepali samples were supplemented with those from Bhutan (N = 19; S2 Table, Fig 2B), collected from four west-to-east drainage basins: Wang Chhu (N = 2); Punatsang Chhu (N = 11); Mangde Chhu (N = 3); Dangme Chhu (N = 3).

Genomic DNA was extracted using the Qiagen DNeasy kit, per manufacturer’s instructions. We utilized published primers to amplify mtDNA *cyt-b* [28], with products enzymatically purified, sequenced using BigDye (ver.3.1) chemistry (Applied Biosystems Inc. [ABI], Forest City, CA), and analyzed on an ABI Prism 3700 Genetic Analyzer (W.M. Keck Center for Comparative and Functional Genomics, University of Illinois, Urbana/Champaign). In addition, we accessed 68 mtDNA *cyt-b* sequences representing 39 species (N = 60 *Schizothorax* plus eight outgroups [*Acrossocheilus* (N = 2); *Pethia* (N = 2); *Puntius* (N = 1); *Tor* (N = 2); *Neolissochilus* (N = 1); S3 Table] (National Institute of Health; https://www.ncbi.nlm.nih.gov/genbank/). To avoid potential ambiguities with regards to evolutionary trajectories, we chose outgroups from select tribes identified as having originated prior to evolution of the Schizothoracini. These include (in sequence): Torini (i.e., *Neolissochilus*); Smilogastrini (i.e., *Puntius*, *Pethia*); and Acrossocheilini (i.e., *Acrossocheilus*, the most immediate outgroup) (S3 Table). Additional details on generating molecular data are provided [29]. Sequences were edited/aligned using Sequencher v.5.3 (Gene Codes Inc., Ann Arbor, MI), with nucleotide composition and uncorrected *p*-distances derived among groups (Mega X; [30]).

### Polyploidy and choice of marker system

The subfamily Cyprininae displays extensive polyploidy (>400 species; [20, 31]), and given the number of polyploid species, different ploidy levels, and the variety of independent polyploidization events identified, it may represent the most complex vertebrate polyploid system currently known [32]. Ploidy is an important consideration when molecular phylogenies are reconstructed in that it significantly impacts statistical approaches, results, and subsequent interpretations. For example, polyploidy complicates the detection of population structure, assignment tests, and principal component analyses [33]. These limitations are particularly problematic given the greater redundancy of nuclear genes in polyploid genomes. All may potentially diverge one from another over evolutionary time, such that results can differ markedly, depending upon which ‘sub-genome’ is being accessed when polyploid data are phylogenetically evaluated [34].

Given this, phylogenetic analyses of polyploid species are often limited in practice to either mtDNA (given its haploid nature), or nuclear genes cloned into bacteria and subsequently isolated as single-copy (such as RAG-1) [20]. However, the latter are evolutionarily more conservative and, for example, cannot be utilized in a TimeTree analysis [35] as herein. Yet polyploidization in *Schizothorax* also represents an evolutionary mechanism that may have facilitated its invasion of high-elevation habitat [31].

We thus restricted our study to mtDNA haplotypes and relied upon GenBank to increase our sample number and geographic representation. Available numbers are often limited, due either to restricted sampling and/or logistic difficulties in accessing those regions where study species occurs. In this context, *Schizothorax* inhabits regions notoriously difficult to access, both topographically as well as socio-politically. In addition, GenBank samples often represent output from ongoing investigations and their metadata thus lack precise GPS coordinates required for specific analyses. This, as well as the large variance in number of base pairs available per sample, has constrained our total GenBank sample number. Many of the above issue can also confound analyses and, to avoid such issues, we rigorously assessed GenBank metadata prior to incorporating sequences.

### Analyses to infer evolutionary history

We employed a four-step approach to infer potential evolutionary scenarios that could explain the biodiversity in *Schizothorax* across the Himalaya. This involved: (a) Evaluating congruence between taxonomic and genetic designation of samples; (b) Detecting cryptic biodiversity; (c) Characterizing regional and basin-specific genetic lineages; and (d) Quantifying phylogeographic patterns. We employed a Bayesian clustering analysis to identify regional gene pools without *a priori* assumptions pertaining to location and/or taxonomic classification. We then used these groupings to statistically evaluate genetic variation within and among regions and basins (AMOVA). We generated phylogenetic hypotheses by deriving ML and Bayesian tree topologies, which were statistically and qualitatively compared for congruence (IQ-tree). We also derived a haplotype network as a means of visualizing the number of stepwise mutations separating distinct sequences and groups. Fossil-calibrated methods were then employed to estimate divergence times for major groupings in the context of regional geologic events.

After compiling a dataset of 132 *Schizothorax* samples using the criteria described above, we derived a dataset that included only variable sites (N = 365bp: DNAsp v.6; [36]). We employed a Bayesian approach (Geneland v.4.0.3: [37, 38]) to infer the number of distinct groups (*K*) and to designate group membership for each sample. Ten runs of 100,000 MCMC iterations and a thinning interval of 100 were used, with *K* varying between 1 and 15, but without use of optional geo-referencing, given the absence of such coordinates for most GenBank sequences. The estimated number of populations (*K*) was defined using the maximum posterior estimate. We conducted an analysis of molecular variance (AMOVA: Arlequin v.3.5.1.2; [39]) to gauge how genetic variation was partitioned statistically among geographic regions (as informed by Geneland). Here, we defined populations (= distinct gene pools) as being haplotypes within regions [40]. Probabilities were ascertained using 1,000 permutations, and statistical significance was assigned using a Bonferroni-correction.

We derived phylogenetic hypotheses using a maximum likelihood (ML) analysis (IQ-Tree v2; [41]), with the best-fitting substitution model being generated via ModelFinder, selecting flexible rate heterogeneity (e.g., gamma-distributed) across sites [42]. Model space was hierarchically explored by sequentially adding parameters (e.g., rate categories) until the model failed to improve. Support for the resulting tree, inferred using the selected model, was explored using 1,000,000 ultrafast bootstrap approximations (UFBoot2; [43]). We also assessed branch support using the SH-aLRT test (Shimodaira-Hasegawa approximate Likelihood Ratio Test, based on a null distribution derived from bootstrap replicates of varying sizes). Values ≥80% indicated strong support for clades, whereas those for UFboot were ≥95% (IQ-Tree v.2 Manual: http://www.iqtree.org).

To employ a Bayesian approach, we first derived an input XML file using a relaxed molecular clock with an uncorrelated lognormal rate distribution and a Yule process prior (graphical user interface (i.e., Beauty) within the phylogenetic software suite Beast (v2.61; [44]). Each analysis was run for 100 million generations, with samples taken every 1,000^th^ iteration, following a burn-in of 20%. Effective sample size (ESS) was verified as >200 across all parameters with MCMC chain convergence visually inspected using Tracer [45]. Trees from three independent runs were then combined (via LogCombiner) as a maximum clade credibility (MCC; TreeAnnotator [44]), with nodal support representing posterior probabilities. The resulting topology was visualized using FigTree (v.1.4.3; [46]).

We statistically compared topologies from ML and BA analyses with respect to the original sequence alignment (IQ-Tree2 v2; [43]). We then examined the support displayed by each regarding RELL approximation (10,000 replicates), as well as: (a) Raw change in log-likelihoods; (b) Bootstrap proportions [47]; (c) Kishino-Hasegawa test [48]; (d) Shimodaira-Hasegawa test [49]; (e) Approximately Unbiased test [50]; and (f) Expected Likelihood weights [51].

### Developing a TimeTree

For molecular dating, we employed a rapid, high-performance ML-based model (RelTime, MEGA X; [33]) that incorporates a relative rate framework (RRF) within which the evolutionary rates of sister lineages are contrasted against a minimum rate change, as diagnosed between evolutionary lineages and their respective descendants. The computational speed and accuracy of the model are appropriate for the analysis of large data sets (>100 ingroup sequences; [52], with procedural steps for RelTime previously documented [53]). The evolutionary model for our ML-based phylogenetic tree was also implemented in RelTime.

We estimated divergence times based on the earliest fossil evidence for Schizothoracini (e.g., *Paleoschizothorax qaidamensis*; [54], as gauged in the Qaidam Basin [55]). This placed initial divergence at the onset of the Oligocene (33 Ma) (see also [56]).

### Phylogeographic analyses

A haplotype network provides a visualization of genealogical relationships based on individual mutations separating haplotypes, and as such, provides insights into genetic diversity within populations or closely related species. It is beneficial in that it effectively separates those individuals with similar haplotypes th generally depicted within a phylogenetic tree as unresolved polytomies (i.e., ’combs’). However, it differs from a strictly bifurcating phylogenetic visualizations in that each haplotype (depicted as a circle) can potentially be connected to multiple, rather than just a single, node.

A second difference is that a comparative phylogenetic tree represents a platform from which genetic variation can be interpreted within the context of geologic history, as supported by statistical and/or Bayesian analyses [57]. In addition, a phylogenetic tree is rooted from ancestral to more derived, with geographically co-distributed species (and their shared lineage histories) transcending. In short, relationships can be well-depicted within a species using a haplotype network, but often much less so for distinct species, given the increase in genetic variability and the lack of an evolutionary trajectory.

**Fig 3:**
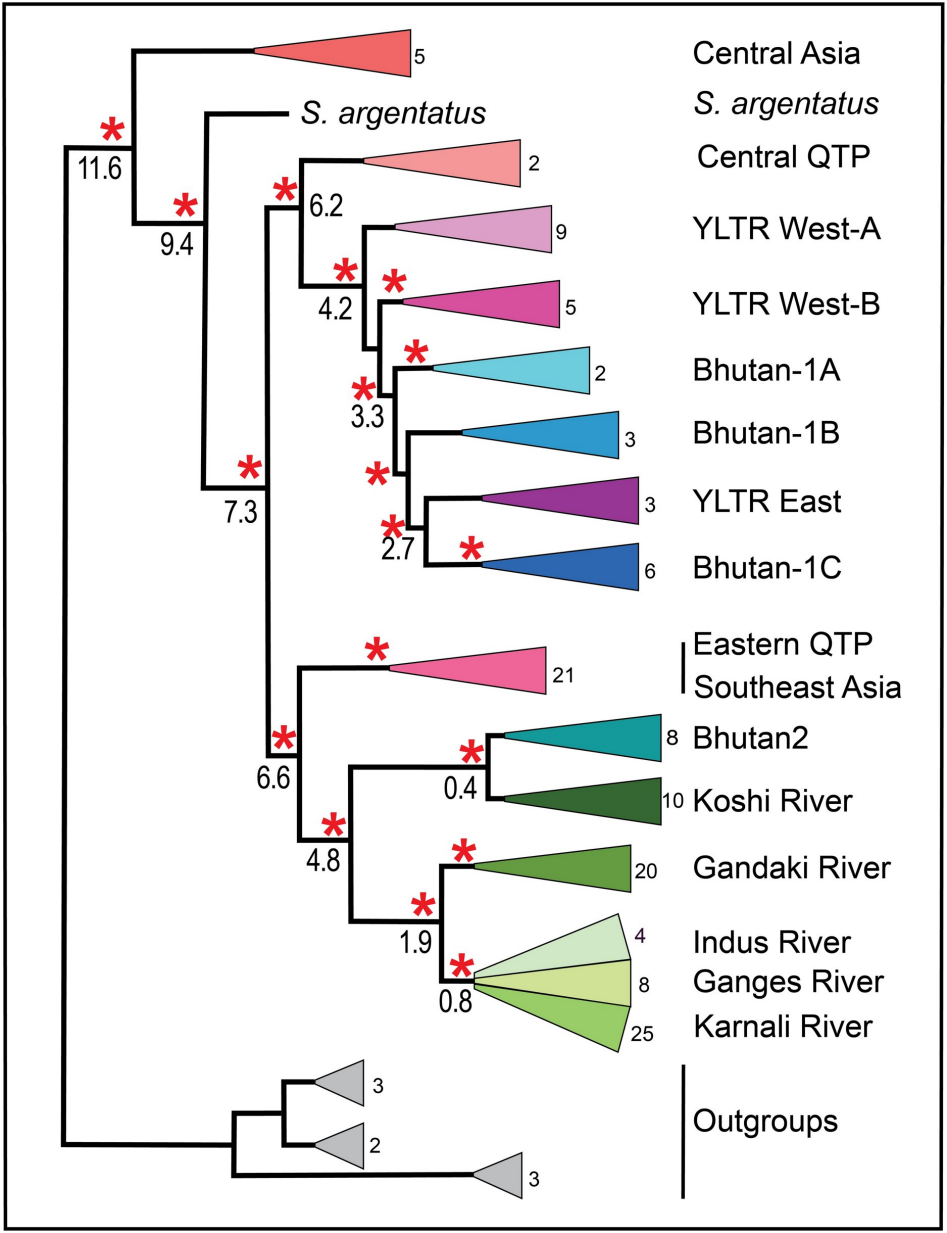
A collapsed maximum likelihood (ML) tree reflecting phylogenetic relationships among biogeographic groups. The constituent samples comprising each biogeographic clade are provided in S3 Table under ’Species Name,’ ’Haplotype,’ ’Accession #,’ ’Location,’ and ’Group’ headings. Data were derived from sequence analysis of the cytochrome-b mitochondrial gene (1,140 bp, 140 haplotypes, clades collapsed). Red asterisk = SH-LRT (Shimodaira-Hasegawa approximate likelihood ratio test) ≥80% and UFboot (ultrafast bootstrap approximations) ≥95% (IQ-Tree v.2 Manual: http://www.iqtree.org). Numbers at colored triangles indicate the number of haplotypes grouped within each. Numbers at nodes represent RelTime Timetree mean divergence estimates (in Ma; confidence intervals in text; See also S1 Appendix).

**Fig 4:**
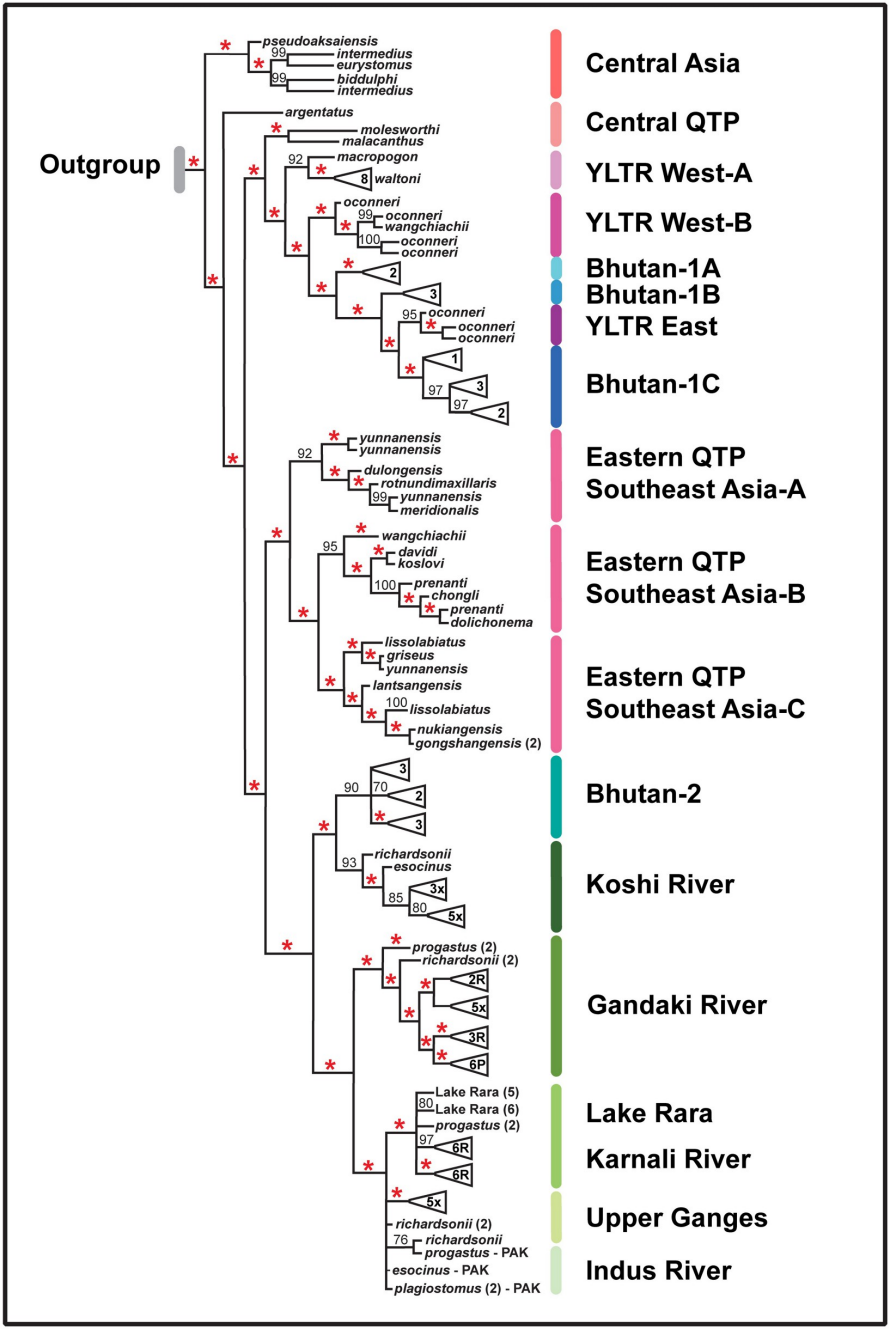
Maximum likelihood (ML) tree reflecting phylogenetic relationships among biogeographic samples. The constituent samples representing each biogeographic clade are provided in S3 Table under ’Species Name,’ ’Haplotype,’ ’Accession #,’ ’Location,’ and ’Group’ headings.’ Data were derived from sequence analysis of the cytochrome-b mitochondrial gene (1,140 bp, 140 distinct haplotypes). Red asterisk = SH-aLRT (Shimodaira-Hasegawa approximate likelihood ratio test) ≥ 80% and UFboot (ultrafast bootstrap approximations) ≥ 95% (IQ-Tree v.2 Manual: http://www.iqtree.org). Numbers only at specific nodes represent UFboot values. Letters within composite species-triangles at tips of the tree represent species, with R = *S*. *richardsonii*, P = *S*. *progastus*, and x = Both species, with numbers representing individuals contained.

We derived a network representing the 132 *Schizothorax* haplotypes using PopART (POPulation Analysis with Reticulate Trees, v.1.7; https://popart.maths.otago.ac.nz/download/ [58]), which employs the TCS metric ([59], as implemented in [60]). To promote visualization, each haplotype was colored per its basin of origin (S3 Table), with colors corresponding to groups depicted in phylogenetic trees (Figs 3 and 4; black circles reflect hypothetical haplotypes not represented in our dataset. Each line indicates a single mutation connecting two haplotypes, but larger numbers of mutational steps are summarized within a black circle).

## Results

### Sequence data

Our phylogenetic analyses incorporated 140 cytochrome-b (*cyt-b*) haplotypes, each spanning 1,140 bp. Of these, 643 sites were monomorphic (= 56%), 432 parsimony-informative (38%), and 65 (6%) represented singletons. Based on the Bayesian Information Criterion (BIC), the best fitting model of sequence evolution was TN+F+I+G4 (i.e., unequal evolutionary rates among purines/pyrimidines, empirical base frequencies, proportion of invariant sites, and a 4-rate category discrete Gamma model).

### *Schizothorax* across Asia and the Himalaya

Our Bayesian approach without *a priori* assumptions detected evidence for 13 discrete, geographically designated groups, (Fig 3). A ’ghost lineage’ emerged as an additional group but was subsequently disregarded as an artifact of isolation-by-distance (per [61]). Groups were designated according to geographic locations of constituent samples. The AMOVA indicated sequence variation was primarily apportioned among the 13 groups, accommodating 78% of the total observed variance. Additionally, all were found to be significantly different one from another (Table 1). Lineages within regions also differed significantly, as did haplotypes within lineages (Fig 4). Probabilities for all comparisons were based on 1,000 permutations, with significance gauged by Bonferroni-correction (*p*<0.005 for 13 regions; *p*<0.0017 for 29 lineages).

**Table 1:** Results of an analysis of molecular variance (AMOVA) based on 132 haplotypes of snowtrout (Cyprinidae: *Schizothorax*). Source represents: (1) Geographic regions (= Among regions); (2) Lineages within regions (= Within regions/among lineages); (3) Haplotypes within lineages (= Within lineages). Source = Grouping; D.F. = Degrees of freedom; S.S. = Sums of squares; Var. = Variance; % = Percent variance. Probability values for each *F*_st_-value (derived via 1,000 permutations) are significant at a Bonferroni-adjusted value of p<0.005.

Source	D.F.	S.S.	Var.	%
Among regions	10	3958.7	31.4	77.9
Within regions/among lineages	18	466.1	5.6	13.5
Within lineages	104	350.6	3.4	8.5
Total	132	4775.3	40.3	99.9

The visualization of phylogenetic relationship among geographic groups and constituents using maximum likelihood (ML) and Bayesian (BA) approaches resulted in largely consistent topologies (ML tree: Figs 3 and 4; Bayesian tree: S1 Fig). Statistical tests of topology subsequently excluded the BA tree (*p*<0.05; Table 2). Given this, our interpretations primarily focused on the ML topology, but also highlighted differences between the two.

**Table 2:** Statistical topology tests comparing maximum likelihood (ML) and Bayesian (BA) phylogenetic results (= Tree). logL = Log Likelihood value; deltaL = logL difference from maximal logL in the set; Bp-RELL = Bootstrap proportions using RELL method (weights sum to 1 across trees); p-KH = *p*-value of one-sided Kishino-Hasegawa test; p-SH = *p*-value of Shimodaira-Hasegawa test; c-ELW = Expected Likelihood Weight (weights sum to 1 across trees); p-AU = *p*-value of Approximately Unbiased test. A plus sign (+) next to a *p*-value denotes 95% confidence sets, whereas a minus sign (-) denotes a significant exclusion (i.e., the tree is rejected). All tests stemmed from 1,000 resamples using the RELL method.

Tree	logL	deltaL	bp-RELL	p-KH	p-SH	c-ELW	p-AU
ML	-9526.83	0.0	0.986 +	0.986 +	1.00 +	0.978 +	0.985 +
BA	-9582.76	55.93	0.014 -	0.013 -	0.013 -	0.014 -	0.015 -

Most nodes in the ML trees were strongly supported by SH-aLRT values and ultrafast bootstrap approximations [e.g., composite (Fig 3) and extended (Fig 4)]. Nodes were likewise supported in the BA tree, with the majority displaying a posterior probability ranging from 0.9–1.0 (S1 Fig).

Two surprising results emerged in both analyses: Bhutanese snowtrout were consistently non-monophyletic, nesting instead within two spatially and temporally disparate clades (i.e., Central QTP/ YLTR *versus* Koshi basin of Nepal). This suggests multiple, independent colonization of high-elevation Bhutanese drainages. In contrast, Nepali snowtrout clustered in a distinct east-to-west pattern, diverging in a sequential manner consistent with the broad-scale topographies of major tributaries, and with sub-clades being approximately congruent from a taxonomic standpoint. The specifics of group membership are provided in S3 Table.

Groups recovered using different genetic clustering approaches corresponded generally with geographic regions (N = 13), as depicted in the ML tree (Fig 3). These are: (1) Central Asia; (2) Central QTP; (3) YLTR West-A; (4) YLTR West-B; (5) Bhutan-1A; (6) Bhutan-1B; (7) YLTR East; (8) Bhutan-1C; (9) Eastern QTP/Southeast Asia-Clade; (10) Bhutan-2 (via Brahmaputra River); (11) Koshi basin (eastern Nepal); (12) Gandaki drainage (central Nepal); (13) a composite western Himalaya clade that included Rara Lake and the Karnali drainage (western Nepal), the upper Ganges (northwest India), and the Indus River Basin (Pakistan).

A more detailed perspective is presented in Fig 4, with regional composites listed in S3 Table. Most noteworthy is the partition of the Eastern QTP/ Southeast Asia (E-QTP/ SEA) clade into three distinct geographic clusters: E-QTP/ SEA-A (Salween-Irrawaddy drainages); E-QTP/ SEA-B (upper Yangtze drainage); (9) E-QTP/SEA-C (Mekong-Salween drainages). In the BA tree (S1 Fig) the western Himalaya was additionally partitioned into two subclades: Indus/Upper Ganges *versus* Karnali/Rara Lake.

In the ML tree, samples from Central Asia formed a distinct regional clade that clusters as sister to all other samples, including. *S*. *argentatus*, which is sister to all remaining species. These form two distinct composite clades. In the first, samples representing the Central QTP form a group, followed by the YLTR West-A and YLTR West-B, then Bhutan-1A separated from Bhutan-1B, and finally a group representing by YLTR East and Bhutan-1C.

The second large ML cluster is subdivided into two distinct sister clades, each partitioned into subclades consistent with geographic regions. The first comprises the Eastern QTP/ Southeast Asia and is represented by three significantly different groups (labeled A, B, C). The second contains the Koshi River (eastern Nepal) as sister to the Bhutan-2 group (representing eight undescribed Bhutanese samples), followed by the Gandaki River as sister to a mixed western Himalaya clade containing the Karnali River/ Rara Lake, the upper Ganges River, and the Indus River (Figs 3 and 4). Clustering of *S*. *richardsonii* and *S*. *progastus* in the Koshi, Karnali, Ganges, and Indus River drainages is noteworthy because individuals do not cluster separately by taxonomic designation within each drainage, but instead group as co-mingled components.

**Fig 5:**
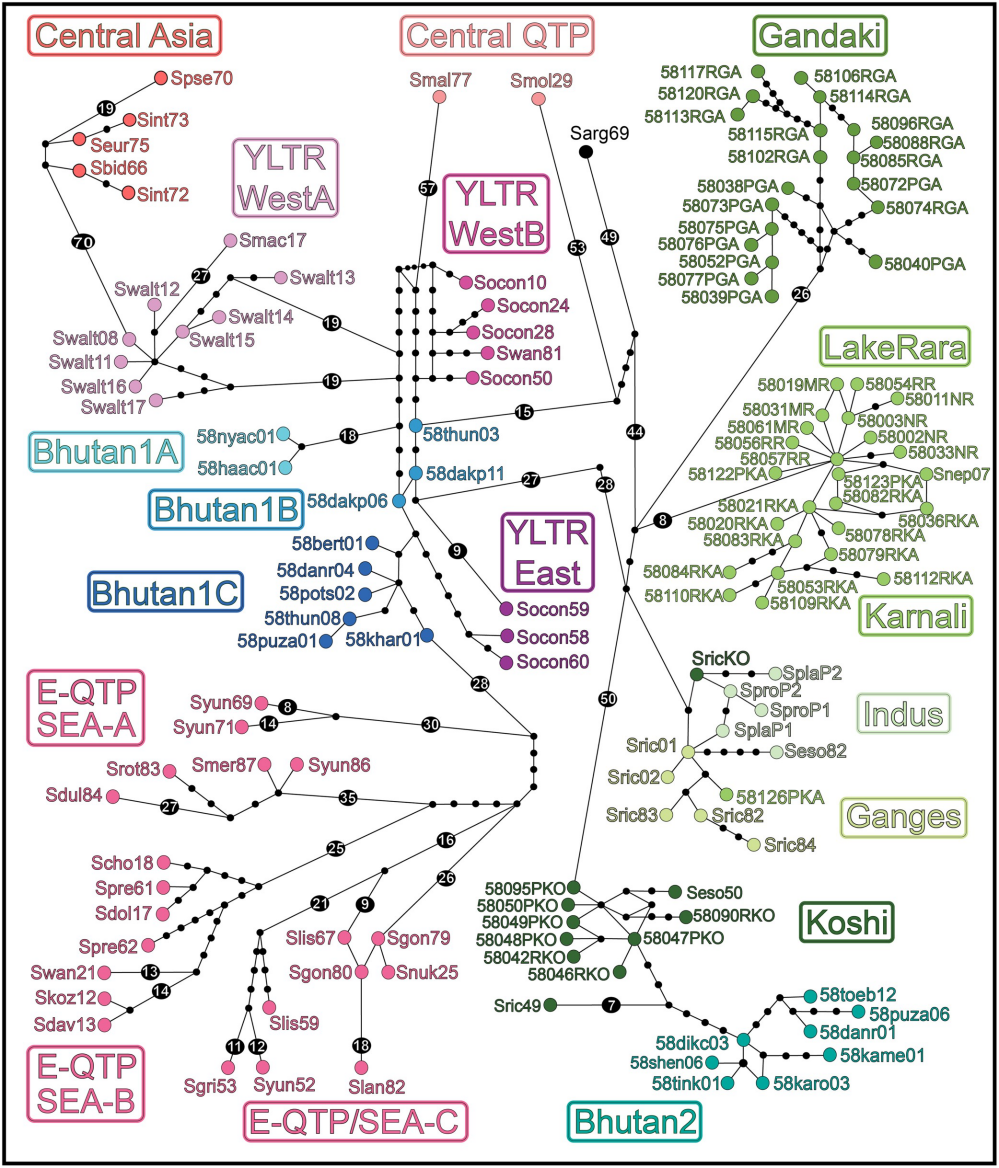
TCS haplotype network reflecting stepwise mutational differences between samples and among geographic groups. The contents of each biogeographic clade are provided in S3 Table under ’Species Name,’ ’Haplotype,’ ’Accession #,’ ’Location,’ and ’Group’ headings. Data were derived from sequence analysis of the cytochrome-b mitochondrial gene (1,140 bp, 132 haplotypes). Geographic groupings (Fig 3, S3 Table) are labeled within rectangles, with group colors identical to those in Figs 3 and 4. Black circles represent hypothetical haplotypes to reflect stepwise mutations >14. See S1 Appendix for the associations between divergence intervals for clades and geomorphic/ climatic drivers in the Himalaya.

Major differences between ML (Figs 3 and 4) and BA (S1 Fig) trees include: Position of the Central Asia and E-QTP/ SEA clades, relationship of YLTR and Bhutan-1 subgroups, and position of *S*. *argentatus* in relation to the Central Asia group. The regions are the same within the BA tree, but distributed across two large regional clusters, with Central Asia included within the first, rather than being distinct from it. YLTR West is subdivided into groups by the presence of Bhutan-1A, whereas Bhutan-1B is instead subsumed within it rather than being sister to YLTR West/Bhutan-1C. The E-QTP/ SEA-A clade is also further subdivided within the BA tree (a pattern also distinct in the haplotype network, Fig 5). And lastly, the Ganges and Indus rivers appear as distinct western Himalaya clades, whereas they are unresolved in the ML tree.

### Temporal divergences

We employed RelTime to derive major time-calibrated events, 13 of which are depicted in our ML-based tree (Fig 3; other dates annotated within discussion; See also S1 Appendix for the associations between divergence intervals and geomorphic/ climatic drivers in the Himalaya).

The average divergence time for Central Asia was 11.6 Ma (CI = 15.4–8.7). Two large subclades sister to Central Asia then separated at ~7.3 Ma (CI = 11.3–4.7). In the first, the Central QTP diverged at 6.2 Ma (10.2–3.7), followed by the YLTR West (4.2 Ma; CI = 7.4–2.4), then Bhutan-1A (3.3 Ma; CI = 6.7–1.5), with YLTR East and Bhutan-1B at 2.7 Ma (CI = 5.9–1.2).

In the second large subclade, the Eastern QTP/ Southeast Asia region separated at 6.6 Ma (CI = 10.7–4.1), followed by a second large composite clade at 4.8 Ma (CI = 8.8–2.6). Here, the Gandaki River/western Himalayan clades subsequently diverged at 1.9 Ma (CI = 3.9–0.9), while Bhutan-2/ Koshi River did so at 0.4 Ma (CI = 0.8–0.2).

### TCS Haplotype network

Geographic groups are also apparent in the haplotype network (Fig 5), and are distinct from one other by at least 19 mutations (numbers in black circle). The network shows fine-scale patterns, with the location of certain haplotypes reflecting inconsistencies between ML and BA trees (e.g., Bhutan-1A,B,C and YLTR East). Some haplotypes display multiple linkages or starburst patterns within the network, a characteristic of recently evolved, within-population genetic diversity (e.g., Rara Lake). In contrast, several other clusters divide into distinct subgroups separated by numerous mutations, and thus reflect deeper phylogenetic divergences (e.g., E-QTP/ SEA-A,B,C).

Haplotypes in Central Asia (top, left) and E-QTP/ SEA (bottom, left) are quite distinct, with each regional group separated by >58 mutations. Clusters representing Bhutan-1 (A,B,C) and YLTR (East, West-A, West-B) are nested in-between.

The Gandaki River (top, right) reflects subgroups generally representing *S*. *progastus* and *S*. *richardsonii* (respectively), while the Karnali River (middle, right) contains a starburst incorporating the three Rara Lake species. Haplotypes from the Indus and the Ganges rivers (middle right) are the most similar and, in turn, reflect geographic groupings with no clear separation. Similarly, Koshi River and Bhutan-2 (lower right) are likewise quite similar, yet clearly distinct from other Western Himalaya haplotypes (>50 mutations).

Our network exhibits two distinct patterns: (a) Clusters of similar haplotypes, such as the Karnali and Gandaki river groups, reflecting scant internal divergence normally characteristic of within/among population genetic diversity; and (b) Groups comprised of considerably divergent haplotypes, such as the E-QTP/ SEA assemblage (*sensu lato*, lower left). The latter is to be expected given the geographic spread of its constituent samples ([21], S2 Table).

## Discussion

### Synergy between tectonism and climate

Orogeny and tectonism drive the diversification of aquatic ecosystems, and act synergistically with climate to impact both hydrology [62] and resident freshwater fishes [63]. For example, historic drought over evolutionary time in western North America gradually shaped tectonically-derived drainage systems, resulting in the erosion of genetic diversity, with populations of endemic fish repeatedly collapsing into refugia then expanding outward during more pluvial periods [64].

Episodic flooding, on the other hand, allowed antecedent streams to down-cut, with headwaters subsequently eroding, basins concomitantly expanding, with large-scale dispersals being promoted [65]. Such alterations (i.e., captures, diversions, beheadings; [66]) not only extended fishes into adjacent basins [67] but also facilitated hybridization [68–70], a process that has (and continues to) confound both taxonomic resolution and management strategies.

Flows within basins are often abridged by longitudinal barriers that are vicariant for larger-bodied species, yet serve as passive filters for fishes smaller and more limited in dispersal capacity [71]. As a result, fish distributions represent a series of biogeographic ’islands’ within and among basins, each reflecting local expansions, contractions, and extinctions. This process provides a necessary template from which to gauge the response of fishes to similar tectonic, orogenic, and climatic impacts within dendritic riverscapes less well monitored (as herein).

The Himalaya provides such a template as shaped by tectonic and climatic events. Its sharp elevational gradients derived via orogeny provided ample opportunities for diversification. For example, many deeply incised valleys emerged along the border of the elevating QTP, and given their increasing gradients, subsequently transitioned into prominent geomorphic features. As a result, many unique physiographic characteristics emerged, particularly when compared with other large global river networks. For example, five (of 10) QTP rivers [i.e., Yellow, upper Yangtze (= Jinsha), upper Mekong (= Lancang), upper Salween (= Nujiang), and Yarlung-Tsangpo (= upper Brahmaputra)] originate at >4,500m elevation, flow >1,000km to the border of the QTP, and subsequently drop >3,000m to Asian lowlands. Interestingly, the Mekong, Salween, and Yangtze flow in parallel for ~170km, and achieve their closest proximity at <70km (Fig 1B; [72]). In S1 Appendix, we provide references that link divergence intervals for our phylogeographic clades with geomorphic/ climatic drivers in the Himalaya.

These events, in turn, have established the region as a global hotspot for biodiversity endemism [21, 73] with Snowtrout as a characteristic element of the aquatic fauna. A more specific example is found within the YLTR, where a deep canyon derived via orogeny effectively partitioned *S*. *oconnori* [74], promoting several distinct ESUs (Evolutionarily Significant Units; [75]). This vicariance similarly impacted *S*. *waltoni* (BA tree; [76]), but those components are evolutionarily less distinct and designated instead as MUs (Management Units; [75]).

Studies of Himalayan freshwater most often focused on select regions, rivers, and/or mountains [77–80]. Our approach broadens that perspective, in that we fill biogeographic gaps in the Himalaya and leverage existing data to more fully evaluate origin and subsequent dispersal of Snowtrout across Asia.

### Central Asia

Five species from Central Asia form a distinct group, but their relationships within *Schizothorax* differs per ML and BA analyses, as does *S*. *argentatus*. In the ML tree (Figs 3 and 4), the Central Asia group clusters as a clade apart from all other *Schizothorax*, with *S*. *argentatus* positioned as sister to all remaining species. In contrast, the BA tree (S1 Fig) places the Eastern QTP/ Southeast Asia as a separate clade outside other species, with the Central Asia group clustering with *S*. *argentatus* as sister to the remaining species. These six species represent the same geographic region (Fig 3) and exhibit potential regional and/or taxonomic substructure, with S. *pseudoaksaiensis* and *S*. *argentatus* distributed in Lake Balkhash and its catchment basin (Kazakhstan, central Asia), as well as the Ili River (northwest China; [81]). Similarly, *S*. *biddulphi* [80] and *S*. *eurystomus* [82] occur in the Tarim River and tributaries (an endorheic basin in the Central Asiatic Desert of northwest China), whereas *S*. *intermediu*s (represented by two distinct haplotypes) has a more extensive range through Iran, Uzbekistan, Kazakhstan, and Kyrgyzstan. Our TimeTree estimation indicated the Central Asia group emerged at 11.6 Ma (CI = 14.3–6.2), an estimate comparable with previous results (11.4–10.5 Ma; [5]). Our estimate for *S*. *argentatus* (9.4 Ma; CI = 14.3–6.2) is also corroborated by earlier estimates (9.6–8.9 Ma; [5]). Based on the results of our ML tree, it seems reasonable to hypothesize Central Asia as the schizothoracine center of origin.

### Central QTP, YLTR, and ’Early Bhutan’

The next major split, at 7.3 Ma (CI = 11.3–4.7), is again consistent with previous estimates (8.3–7.6 Ma) and defines two large groups (Figs 3 and 4). The first encompasses species of the Central QTP, Bhutan (immediately south), and the YLTR. The second is split into two sister clades: One represents species from eastern QTP/ Southeast Asia, whereas the second incorporates Himalayan species hypothesized to have dispersed westward via the Brahmaputra River.

The two Central QTP species (*S*. *molesworthi and S*. *malacanthus*) separated in early Miocene (6.2 Ma; CI = 10.2–3.7), whereas the YLTR West diverged late Pliocene (4.2 Ma; CI = 7.3–2.4). The YLTR-West species cluster as two separate groups, with *S*. *macropogon* (3.2 Ma; CI = 6.6–1.5, late Pliocene) as sister to *S*. *waltoni* (YLTR West-A), which subsequently diverged into western and eastern groups detectable via microsatellite DNA but not *cyt-b* [76]. These species are sister to a second geographically structured group (YLTR West-B) comprised of *S*. *oconnori and S*. *wangchiachii*, which clusters as sister group to samples from Bhutan and YLTR East.

The evolutionary history of *S*. *oconnori* (endemic to the YLTR; [81]) underscores a late Miocene divergence driven by QTP uplift. During this process, the development of a precipitous canyon [72] separated *S*. *oconnori* into distinct western and eastern components [74]. The results of our composite analyses (SH-aLRT, UFboot, AMOVA) sustain this hypothesis, with the western group (to include *S*. *wangchiachii*) differing significantly from eastern *S*. *oconnori* (YLTR-East), and separated from it by two undescribed Bhutanese lineages (i.e., Bhutan-1A, Bhutan-1B). Interestingly, a third Bhutanese lineage (Bhutan-1C) clusters with YLTR-East. We consider these three as representing ‘Early Bhutan,’ given their divergence from a fourth Bhutanese clade (Bhutan-2) that apparently originages from a more recent dispersal event (Fig 3).

Similarly, separate dispersals seemingly occurred for *S*. *oconnori* in the ancestral YLTR. One into western Bhutan (Bhutan-1A), and a subsequent one into central/eastern Bhutan (Bhutan-1B,C). Each is currently unverified as to species but can be tentatively allocated to *S*. *oconnori* (i.e., as *Schizothorax sp*. *cf oconnori*). It thus appears as if Quaternary climatic oscillations not only yielded distinct evolutionary groupings in both *S*. *waltoni* and *S*. *oconnori*, but also among *Schizothorax* represented in Bhutan (S1 Appendix). Again, the BA-tree differs in placement of the YLTR and Bhutan-1 lineages, with the YLTR-West-A and YLTR West-B groups separated by the Bhutan-1A group, whereas Bhutan-1B and Bhutan-1C groups collapse and YLTR-East placing as sister to it (S1 Fig).

### Eastern QTP and Southeast Asia

The E-QTP/ SEA-A species split into three significantly different clades (Fig 4, S3 Table), encompassing five species from the Irrawaddy/ Salween rivers and reflecting the deepest TimeTree estimate at 5.6 Ma (CI = 9.7‒3.0). The E-QTP/ SEA-B diverged at 1.7 Ma (CI = 3.5–0.8) and consists of seven species from the Yangtze River, whereas the E-QTP/ SEA-C involves seven species from the Mekong/ Salween drainages. The Mekong divergence was seemingly driven by late Pliocene orogenesis [77], an estimate congruent with our timeline (2.4 Ma; CI = 5.0‒1.2). Three species within the upper Salween (i.e., *S*. *gongshanensis*, *S*. *lissolabiatus*, *S*. *nukiangensis*) display low genetic divergence and frequent haplotype sharing, a potential by-product of Quaternary glaciations [79]. However, several E-QTP/ SEA-B species (such as *S*. *davidi*, *S*. *koslovi*, *S*. *chongli*, *S*. *gongshanensis*) have yet to be fully described or documented, other than in general terms [80].

### Dispersal into Bhutan, Nepal, and western Himalaya

The YLTR originates on the QTP, flows eastward then abruptly south through the Namche Barwa Syntaxis [5], with subsequent capture by the Brahmaputra River [6]. It represents an important freshwater dispersal route from the QTP and its drainage history correlates with an hypothesized evolutionary transition between species on the QTP (*sensu stricto*) *versus* more recent divergences via the Lower Brahmaputra.

In our analyses, samples from Nepal split into distinct clades, each representing separate drainages that are significantly different from Southeast Asian clades. Interestingly, a set of samples from Bhutan (Bhutan-2; N = 8) form a distinct clade sister to the easternmost Nepali drainage (Koshi River Basin; N = 10, S3 Table). This is consistent with an hypothesized east-to-west dispersal via the lower Brahmaputra. Species in the Koshi River group are tentatively referred to as *S*. *richardsonii*, *S*. *esocinus*, and *S*. *progastus*, whereas samples from Bhutan-2 are currently unidentified but link in the network with a haplotype of *S*. *progastus* from the Koshi River (Fig 5).

The morphological similarities between *S*. *richardsonii* and *S*. *progastus* in the Koshi, Karnali, and Gandaki rivers are such that their discrimination can be problematic in the field [83], particularly at early life history stages. Despite morphological differences [29], the two species are genetically more similar within than among basins, which could be driven by ecological diversification along elevational gradients, allowing for emergence of similar phenotypes in each basin (i.e., homoplasy). This perspective stems from mtDNA data, but a recent study using ddRADseq indicated substantial admixture between the two in each drainage [84]. Our study does not (and cannot) revise taxonomy, but it does identify regions (e.g., Nepal, Bhutan) where more focused research is needed to assess genetic and phenotypic diversity, and thus clarify taxonomy of *Schizothorax* in the Central and Eastern Himalaya.

*Schizothorax* in the Western Himalaya are genetically less well defined and their clustering reflects a mosaic of historic and contemporary drainage arrangements (S1 Appendix). Samples from the Gandaki and the Karnali rivers cluster into distinct groups, with the Gandaki River haplotypes (N = 20) most distinct (separated by 26 mutations, Fig 5) and partitioned into nested clusters of *S*. *richardsonii* (three lineages), S. *progastus* (two lineages), and one representing a mixture of the two (Fig 4). Samples from the Karnali River Basin (far western Nepal, N = 25) fall within a composite that encompasses samples from the Upper Ganges (N = 8) and Indus rivers (N = 4; western Himalaya). These generally group by basin in the BA tree (S1 Fig) yet are weakly separated in the haplotype network (Fig 5), where haplotypes from the Karnali River Basin form a distinct cluster, separated by 11 mutations from the Indus and Ganges (Fig 5). Samples from the Ganges and Indus River basins were designated as *S*. *progastus* and *S*. *plagiostomus*, with *S*. *richardsonii* also identified in the Upper Ganges, and *S*. *esocinus* in the Indus River (S3 Table). The BA tree topology separates the Indus and Ganges and places this group as sister to the distinct Karnali River/ Rara Lake group. These groupings are congruent with contemporary drainage arrangements of the Koshi, Gandaki, and Karnali rivers which flow into the Ganges River of India (Fig 2A) to ultimately drain into the Bay of Bengal. In contrast, the contemporary Indus River drains westward to the Arabian Sea, but the close association of haplotypes between Indus, Upper Ganges and Karnali rivers suggest an alternate historic drainage arrangement.

### Phylogeography of Rara Lake, Nepal

Three morphologically differentiated and reproductively isolated *Schizothorax* (i.e., *S*. *macrophthalmus*, *S*. *nepalensis*, *S*. *raraensis*) occupy separate trophic niches in Rara Lake (Karnali River Basin, northwestern Nepal: [85]), with *S*. *raraensis* and *S*. *nepalensis* listed as critically endangered (IUCN, 2017; http://www.iucnredlist.org/details/168564/0/). Of the three, only *S*. *nepalensis* could be differentiated using mtDNA, and was hypothesized [86] as sharing mtDNA haplotypes with *S*. *macrophthalmus* and *S*. *raraensis* via hybridization. Similarly, two morphologically distinct Snowtrout in the upper Karnali River (i.e., *S*. *richardsonii* and *S*. *progastus*) could not be discriminated, again hypothesized as interspecific hybrids [86]. Our mitochondrial marker could not resolve this hypothesis, thus necessitating further nuclear analyses that may unequivocally test the role of hybridization as a source of phylogenetic discordance. Such an examination, however, is again complicated by polyploidy and its variance within *Schizothorax*. Haplotypes of Rara Lake species are generally distinct, but not clearly separated from each other or from Karnali River samples (Fig 5).

The geologic age of Rara Lake is estimated at ~60 kya [87]. Its endemic species do not form a monophyletic clade but cluster instead with samples from tributaries of the Karnali River which drains the lake (Fig 5, S1 Fig). Haplotypes of *S*. *richardsonii* and *S*. *progastus* do not differ significantly from one another in the Karnali River Basin, but do so from conspecifics in the Koshi and Gandaki rivers [86], a pattern also confirmed in our analyses. Species in the Karnali River Basin are poorly resolved with respect to mitochondrial divergence, with a genetic signal likely obscured by the limited timeframe within which ancestral alleles could sort (i.e., incomplete lineage sorting). More refined molecular approaches, such as reduced-representation genomics [68–70, 88, 89], could potentially provide the necessary resolution, although it will again be compounded by polyploidy, as characterized within Cyprininae.

### Dispersal into western Himalaya tributaries

The southern QTP and western Himalaya are now drained by the contemporary Indus River, which extends south and west from the Karakorum Range rather than from the High Himalaya. Given its western placement, the geomorphic history of the Indus River has an interesting relationship with the biogeography of Himalayan *Schizothorax*. Samples from the Indus and the Ganges (immediately to the east) are unsettled, as reflected in our analyses, with several hypotheses proposed [90]. Relative stasis between the two river basins is the most conservative, whereas others involve various stream-capture scenarios, such as: The Ganges initially flowing westward to the Indus, then to the Arabian Sea; The Indus flowing eastward into the Ganges, then to the Bay of Bengal; and lastly, an earlier capture by the Ganges of four western Himalaya rivers (i.e., Jhelum, Chenab, Ravi, Sutlej) which are now incorporated within the contemporary Indus Basin.

The latter hypothesis is supported by isotopic and seismic data extracted from the detrital fan of the Indus River, as deposited for >30 Ma in the Arabian Sea. These data document a major shift at ~5 Ma, with earlier Himalayan deposition superseding those from the Indus suture zone to the north [91]. This documents the capture of several large Punjabi rivers (i.e., Jhelum, Chenab, Ravi, Sutlej) by the Indus, as represented in the current basin topography. However, this interpretation is not without controversy. A subsequent comparison of sediments from northern Pakistan with those of the Indus Fan suggests the transition was a potential response to differential erosion rates in the source region (the Indus Basin) rather than drainage rearrangements [90].

The east-west divergence among Himalayan clades provides a framework by which the geomorphic evolution of the drainage system can be interpreted. In this sense, our phylogeographic results can be inferred as biologically corroborating the capture by the Indus of those drainages previously allocated to the Ganges Basin. For example, *Schizothorax* from the Lower Ganges River diverged from a lineage representing the Upper Ganges and the Indus River. Similar patterns have been noted for Indus and Gangetic dolphin [92]. These data also suggest *Schizothorax* in the western Himalaya (i.e., Koshi, Gandaki, and Karnali rivers) were isolated prior to the capture of the Jhelum by the Indus River. Overall, our analyses reflect these unresolved questions, as indicated by placement of individual haplotypes apart from others with the same geographic origin (Figs 4 and 5, S1 Fig). A more detailed study is needed as our analyses only include a limited number of samples representing this biogeographic region.

### Taxonomic uncertainty in *Schizothorax* and its implications

Several species in our analyses were polyphyletic, with individuals forming biogeographically defined clusters that differed significantly one from another. In addition, multiple cases also emerged where samples within the same region failed to cluster with conspecifics (e.g., Central Asia; Eastern QTP/Southeast Asia; Koshi; Gandaki; Karnali; Ganges).

Incongruence between molecular and morphological phylogenies represents a challenge for the current study, as reflected by morphologically-identified species from different drainages failing to cluster together. In contrast, the converse is true for different species in the same drainage [16, 78]. This can reflect incomplete lineage sorting [21, 77]; hybridization (intergeneric: [5, 93], intrageneric: [94]); rapid diversification [5]; and/or convergent evolution (e.g., juxtaposition of trophic and spatial niches across species; [95, 96]). None can be resolved using mtDNA [22]. Unfortunately, these issues are amplified within online databases by an incorrect association of DNA sequences with taxonomy [97].

## Conclusions

*Schizothorax* in the Himalaya is a monophyletic genus hypothesized as originating in Central Asia and subsequently dispersing southward into Bhutan via down-cutting rivers, as well as westward across the Eastern QTP and into Southeast Asia. The subsequent capture of the YLTR by the Brahmaputra allowed for an east-to-west dispersal south of the Himalaya, facilitating sequential movements northward into Himalaya drainages, first in Bhutan, then Nepal, and eventually into the western Himalaya. Our molecular data corroborate western drainages as being previously affiliated with the Ganges River but subsequently captured by the Indus River.

Many morphologically-recognized species have also diverged substantially across basins, suggesting the potential for cryptic speciation and/or undescribed, relictual biodiversity. More detailed molecular studies are needed to test this hypothesis and thus promote conservation planning at the regional scale. This will require the analysis of populations now geographically isolated across the Himalaya, with cooperation/ collaboration needed among neighboring countries for comprehensive, large-scale studies [98, 99]. This is particularly imperative given extant (as well as predicted) impacts of climate change on the Third Pole in general, and *Schizothhorax* in particular [26].

## Supporting information

S1 Appendix Divergence intervals (TIMETREE, MEGA X [30]) for Snowtrout (Cyprininae: *Schizothorax*), as associated with geomorphic and climatic drivers in the Himalaya. Confidence intervals for divergence estimates are provided as parentheticals.
(PDF)

S1 Table *Schizothorax* samples from Nepal (N = 53) by site and species. Site = sampling site (N1-N9); Species = *Schizothorax* sp. identification; River = basin; Location = collection area within basin (confl = confluence); Lat = Latitude; Long = Longitude; KU-T = Univ. Kansas Tissue number (KU:KUIT:#); KU-V = Univ. Kansas Voucher number (KU:KUIT:#); Haplotype = Univ. Arkansas Sequence number; Accession = GenBank Accession number. Sites depicted topographically in Fig 2A.
(PDF)

S2 Table *Schizothorax* samples from Bhutan (N = 19) by site and species. Site = sampling site (B1-N9); Species = Schizothorax sp. identification; River = basin; Location = collection area within basin (Chhu = river); Lat = Latitude; Long = Longitude; KU-T = Univ. Kansas Tissue number (KU:KUIT:#); KU-V = Univ. Kansas Voucher number (KU:KUIT:#); Haplotype = Univ. Arkansas Sequence number; Accession = GenBank Accession number. Sites depicted topographically in Fig 2B.
(PDF)

S3 Table Samples of *Schizothorax* (N = 132) and outgroup species (N = 8) evaluated. Species Name = Scientific name (A = *Acrossocheilus*; Pe = *Pethia*; Pu = *Puntius*; T = *Tor*; N = *Neolissochilus*; S = *Schizothorax*); Haplotype = Univ. Arkansas sequence identifier [i.e., from GenBank = letters and numbers; Univ. of Arkansas = starting with ’58’ (Univ. of Kansas tissue vouchers referenced in S1 Table)]; Location = Country, geographic region or river basin; Group = Regions; Group abbreviations are as follows: QTP = Qinghai-Tibetan Plateau; SEA = Southeastern Asia; YLTR = Yarlung-Tsangpo River; * = indicates samples that cluster with a group outside their sampling region.
(PDF)

S1 Fig Phylogenetic tree derived using Bayesian methodology (BEAST v2.61). Phylogenetic relationships among snowtrout (*Schizothorax*; Cyprinidae) and outgroup taxa derived from sequence analysis of the *cytochrome-b* mitochondrial gene (1,140 bp, 140 haplotypes). Individuals are coalesced into geographic regions, with abbreviations defined as: E-QTP/ SEA = Eastern Qinghai-Tibetan Plateau/ Southeast Asia, with three subclades indicated by A, B, and C; YLTR = Yarlung-Tsangpo River, with subclades indicated by West-A, West-B, and East. Numbers at nodes represent Bayesian posterior probabilities. Sample metadata in S3 Table.
(PDF)
