# Supplementary material for: The Himalayan uplift and evolution of aquatic biodiversity across Asia: Snowtrout (Cyprininae: Schizothorax) as a test case
Source: PLoS One. 2023 Oct 24;18(10):e0289736. doi: 10.1371/journal.pone.0289736 (PMC10597529; doi:10.1371/journal.pone.0289736)
Supplement: S1 Appendix — Confidence intervals for divergence estimates are provided as parentheticals. (PDF) [file pone.0289736.s001.pdf]

S1 Appendix. Divergence intervals (TimeTree, Mega X [30]) for *Sno*wtrout (Cyprininae: *Schizothorax*), as associated with geomorphic and climatic drivers in the Himalaya. Confidence intervals for divergence estimates are provided as parentheticals.

Impacts ~34 MA: A remarkable transition from dry to wet conditions occurred at the Eocene/Oligocene boundary (34 Ma). At this time, the Tibetan Plateau seemingly reached an elevation threshold with intensifying monsoonal rains, as gauged by a strong seasonality in gastropod growth rings (a typical monsoonal feature). This seemingly represent orographic impacts of the Himalaya and Tibetan Plateau uplift on global atmospheric circulation [101].

> We estimated divergence times based on the earliest fossil evidence for Schizothoracini (e.g., *Paleoschizothorax qaidamensis*; [54], as gauged in the Qaidam Basin [55]). This placed initial divergence for Schizothoracini at the onset of the Oligocene (~33 Ma) (see also [56]).

Impacts ~15 MA: Yarlung-Tsangpo River captured by Brahmaputra River (~18–15ma: [6, 8]).

> Separation of Central Asia Clade: 11.6 Ma (15.4-8.7).

Impacts ~10 MA: A sharp increase in the rate of erosion occurred during Late Miocene in the Greater Himalaya. It was manifested by elevated sedimentation rates and abrupt changes in geochemical and isotopic composition [102, 103]. This rapid exhumation postdates integration of the Yarlung-Tsangpo and Brahmaputra rivers by at least ∼10 my, with tectonic uplift rather than river capture as the driving agent.

> Separation of *S. argentatus* and wo large Central Asian subclades: 9.4 Ma (14.3–6.2).

Impacts ~6 MA: The paleo-Brahmaputra river was redirected north and west by the elevation of the Shillong plateau at 5.2–4.9 Ma, in response to stresses caused by the Indian lithosphere bending beneath the Himalaya [8]. Also, late Miocene cooling gave way to the early-mid-Pliocene Warm Period (Miocene-Pliocene Boundary, MPB=5.3 Ma. This resulted in a distinct summer monsoon and its subsequent intensification that impacted the Asian hydrologic gradient (East Asia more moist, Central Asia more arid, with accompanying biogeographic and evolutionary implications [104]).

> Central QTP: 6.2 Ma (10.2-3.7).
> Eastern QTP/ SEA: 6.6 Ma (10.7-4.1) and 4.8 Ma (8.8-2.6).

Impacts ~3-2 MA: The increase in erosion rates within Bhutan associated with a surface uplift < 3Ma, and potentially younger than 1.75 Ma [105].

> Bhutan-1A,B,C, YLTR East: 3.3 Ma (5.9-1.2).
> Gandaki River/western Himalaya: 1.9 Ma (3.9-0.9).

Impacts ~1 MA: Reconstructed paleo-river profiles and landscape evolution simulations indicate that low-relief landscapes in Bhutan were uplifted ~800m in the past ~1–0.8 Ma [106]. In addition, a synchronous exhumation pulse occurred in both eastern and western syntaxes of the Himalaya. This suggests in turn that larger-scale tectonic processes in the western Himalaya [Namche Barwa massif (NBM)] that began ~1Ma are primary triggers for the rapid tempo of exhumation that occurred, rather than erosion by rivers and/or glaciers [107].

Separation of Bhutan-2 and the Koshi River: 0.4 MA (0.8-0.2).

Citations [6]–[56] are also provided in References, main text); [101–107] only provided herein.

[6] Bracciali L, Najman Y, Parrish RR, Akhter SH, Millar I. The Brahmaputra tale of tectonics and erosion: Early Miocene river capture in the Eastern Himalaya. Earth Planet Sci Lett. 2015; 415: 25–37. https://doi.org/10.1016/j.epsl.2015.01.022.

[8] Govin G, Najman Y, Copley A., Millar I, van der Beek P, Huyghe P, et al. Timing and mechanism of the rise of the Shillong Plateau in the Himalayan foreland. Geology. 2018; 46: 279–282. https://doi.org/10.1130/G39864.1.

[30] Kumar S, Stecher G, Li M, Knyaz C, Tamura K. Mega X: Molecular evolutionary genetics analysis across computing platforms. Mol Biol Evol. 2018; 35: 1547–1549. https://doi.org/10.1093/molbev/msy096

[55] Guo J, Wei X, Long G, Wang B, Fan H, Xu S. Three-dimensional structural model of the Qaidam Basin: Implications for crustal shortening and growth of the northeast Tibet. Open Geosci. 2017; 9: 174–185. https://doi.org/10.1515/geo-2017-0015.

[56] Zhang D, Yu M, Hu P, Peng S, Liu Y, Li W, et al. Genetic adaptation of Schizothoracine fish to the phased uplifting of the Qinghai-Tibetan Plateau. G3. 2017; 7: 1267. https://doi.org/10.1534/g3.116.038406.

[101] Zheng H, Yang Q, Cao S, Clift PD, He M, Kano A, et al. From desert to monsoon: Irreversible climatic transition at ~ 36 Ma in southeastern Tibetan Plateau. Prog Earth Planet Sci. 2022; 9: 12. https://doi.org/10.1186/s40645-022-00470-x.

[102] Lang KA, Huntington KW, Burmester R, Housen B. Rapid exhumation of the eastern Himalayan syntaxis since the late Miocene. Geol Soc Am Bull. 2016; 128 (9-10): 1403–1422. https://doi.org/10.1130/B31419.1.

[103] Chen W-H, Yan Y, Clift PD, Carter A, Huang C-Y, Pickering KT, et al. Drainage evolution and

exhumation history of the eastern Himalaya: Insights from the Nicobar Fan, northeastern Indian Ocean. Earth Planet Sci Lett. 2020; 548: 116472. https://doi.org/10.1016/j.epsl.2020.116472.

[104] Ao H, Rohling EJ, Zhang, R, Roberts AP, Holbourn AE, Ladant J-B, et al. Global warming-induced Asian hydrological climate transition across the Miocene–Pliocene boundary. Nat Commun. 2021; 12: 6935. https://doi.org/10.1038/s41467-021-27054-5.

[105] Adams BA, Hodges KV, Whipple KX, Ehlers TA, van Soest MC, Wartho J. Constraints on the tectonic and landscape evolution of the Bhutan Himalaya from thermochronometry. Tectonics. 2015; 34: 1329–1347. https://doi.org/10.1002/2015TC003853.

[106] Adams BA, Whipple KX, Hodges KV, Heimsath AM. In situ development of high-elevation, low-relief landscapes via duplex deformation in the Eastern Himalayan hinterland, Bhutan. J Geophys Res Earth Surf. 2016; 121: 294–319. https://doi.org/10.1002/2015JF003508.

[107] Guevara VE, Smye AJ, Caddick MJ, Searle MP, Olsen T, Whalen L, et al. A modern pulse of ultrafast exhumation and diachronous crustal melting in the Nanga Parbat Massif. Sci Adv. 2022; 8: eabm2689. https://doi.org/10.1126/sciadv.abm268.
