# Supplementary material for: The Himalayan uplift and evolution of aquatic biodiversity across Asia: Snowtrout (Cyprininae: Schizothorax) as a test case
Source: PLoS One. 2023 Oct 24;18(10):e0289736. doi: 10.1371/journal.pone.0289736 (PMC10597529; doi:10.1371/journal.pone.0289736)
Supplement: S1 Table — Site = sampling site (N1-N9); Species = Schizothorax sp. identification; River = basin; Location = collection area within basin (confl = confluence); Lat = Latitude; Long = Longitude; KU-T = Univ. Kansas Tissue number (KU:KUIT:#); KU-V = Univ. Kansas Voucher number (KU:KUIT:#); Haplotype = Univ. Arkansas Sequence number; Accession = GenBank Accession number. Sites depicted topographically in Fig 2A. (PDF) [file pone.0289736.s002.pdf]

S1 Table. *Schizothorax* samples from Nepal (N=53) by site and species.

Site= sampling site (N1-N9); Species= *Schizothorax* sp. identification; River= basin; Location= collection area within basin (confl.=confluence); Lat= Latitude; Long= Longitude; KU-T= Univ. Kansas Tissue number (KU:KUIT:#); KU-V= Univ. Kansas Voucher number (KU:KUIT:#); Haplotype= Univ. Arkansas Sequence number; Accession= GenBank Accession number. Sites depicted geographically in Fig 3.

Site	Species	River	Location	Lat	Long	KU-T	KU-V	Haplotype	Accession
N1	*S. progastus*	Karnali	Jhugala	29.5217000	81.7800000	1940	27884	58122PKA	
N1	*S. progastus*	Karnali	Jhugala	29.5217000	81.7800000	1941	27884	58123PKA	
N1	*S. progastus*	Karnali	Jhugala	29.5217000	81.7800000	1944	24884	58126PKA	
N1	*S. richardsonii*	Karnali	Jhugala	29.5217000	81.7800000	1947	27809	58082RKA	MW479778
N1	*S. richardsonii*	Karnali	Jhugala	29.5217000	81.7800000	1948	27809	58083RKA	MW479804
N1	*S. richardsonii*	Karnali	Jhugala	29.5217000	81.7800000	1949	27809	58084RKA	MW479803
N2	*S. richardsonii*	Karnali	Srikot	29.5216999	81.9300003	1935	27810	58020RKA	MW479810
N2	*S. richardsonii*	Karnali	Srikot	29.5216999	81.9300003	1936	27810	58036RKA	MW479818
N2	*S. richardsonii*	Karnali	Srikot	29.5216999	81.9300003	1937	27810	58021RKA	MW479809
N2	*S. richardsonii*	Karnali	Srikot	29.5216999	81.9300003	1938	27810	58078RKA	MW479807
N2	*S. richardsonii*	Karnali	Srikot	29.5216999	81.9300003	1939	27810	58079RKA	MW479806
N3	*S. richardsonii*	Karnali	Gumgarh	29.5533009	82.1699982	1999	27885	58109RKA	MW479787
N3	*S. richardsonii*	Karnali	Gumgarh	29.5533009	82.1699982	2000	27885	58110RKA	MW479802
N3	*S. richardsonii*	Karnali	Gumgarh	29.5533009	82.1699982	2002	27885	58112RKA	MW479800
N3	*S. richardsonii*	Karnali	Gumgarh	29.5533009	82.1699982	2003	27885	58053RKA	MW479808
N4	*S. nepalensis*	Karnali	Rara Lake	29.5333004	82.0667038	1793	27811	58002NR	MW479846
N4	*S. nepalensis*	Karnali	Rara Lake	29.5333004	82.0667038	1794	27811	58003NR	MW479814
N4	*S. macrophthalmus*	Karnali	Rara Lake	29.5333004	80.0667038	1903	27813	58031MR	MW479836
N4	*S. nepalensis*	Karnali	Rara Lake	29.5333004	82.0667038	1906	27811	58011NR	MW479842
N4	*S. nepalensis*	Karnali	Rara Lake	29.5333004	82.0667038	1917	29235	58033NR	
N4	*S. macrophthalmus*	Karnali	Rara Lake	29.5333004	80.0667038	1933	29237	58019MR	MW479837
N4	*S. raraensis*	Karnali	Rara Lake	29.5382996	82.0766983	2004	27814	58054RR	MW479855
N4	*S. raraensis*	Karnali	Rara Lake	29.5382996	82.0766983	2006	27814	58056RR	MW479854

N4	*S. raraensis*	Karnali	Rara Lake	29.5382996	82.0766983	2007	27814	58057RR	MW479853
N4	*S. macrophthalmus*	Karnali	Rara Lake	29.5333004	80.0667038	2011	27813	58061MR	MW479833
N5	*S. richardsonii*	Gandaki	Kali Gandaki	28.6840972	83.6123389	1959	29050	58085RGA	MW479798
N5	*S. richardsonii*	Gandaki	Kali Gandaki	28.6840972	83.6123389	1962	29050	58088RGA	MW479796
N6	*S. richardsonii*	Gandaki	Kali Gandaki	28.4000000	83.5667000	1987	27887	58102RGA	MW479791
N6	*S. richardsonii*	Gandaki	Kali Gandaki	28.4000000	83.5667000	1991	27887	58106RGA	MW479788
N6	*S. progastus*	Gandaki	Kali Gandaki	28.3999996	83.5667038	1995	27806	58052PGA	MW479771
N6	*S. progastus*	Gandaki	Kali Gandaki	28.3999996	83.5667038	8814	27806	58072PGA	MW479770
N6	*S. richardsonii*	Gandaki	Kali Gandaki	28.4000000	83.5667000	8817	27887	58113RGA	MW479786
N6	*S. progastus*	Gandaki	Rahughat	28.3999996	83.5667038	8822	27806	58075PGA	MW479769
N6	*S. progastus*	Gandaki	Rahughat	28.3999996	83.5667038	8824	27806	58076PGA	MW479768
N6	*S. richardsonii*	Gandaki	Kali Gandaki	28.4000000	83.5667000	8825	27887	58114RGA	MW479785
N6	*S. richardsonii*	Gandaki	Kali Gandaki	28.4000000	83.5667000	8826	27887	58115RGA	MW479784
N6	*S. richardsonii*	Gandaki	Kali Gandaki	28.4000000	83.5667000	8828	27887	58117RGA	MW479783
N6	*S. progastus*	Gandaki	Rahughat	28.3999996	83.5667038	8829	27806	58077PGA	MW479767
N6	*S. richardsonii*	Gandaki	Kali Gandaki	28.4000000	83.5667000	8832	27887	58120RGA	MW479780
N7	*S. progastus*	Gandaki	Kali Gandaki-Beni	28.3416996	83.5682983	1955	29043	58038PGA	MW479775
N7	*S. progastus*	Gandaki	Kali Gandaki-Beni	28.3416996	83.5682983	1956	29043	58039PGA	MW479774
N7	*S. progastus*	Gandaki	Kali Gandaki-Beni	28.3416996	83.5682983	1957	29043	58040PGA	MW479776
N7	*S. richardsonii*	Gandaki	Kali Gandaki	28.3403556	83.5692278	1979	27886	58096RGA	MW479794
N7	*S. progastus*	Gandaki	Kali Gandaki	28.3416996	83.5682983	8815	27815	58073PGA	
N7	*S. richardsonii*	Gandaki	Kali Gandaki	28.3416996	83.5682983	8820	27886	58074RGA	MW479799
N8	*S. progastus*	Koshi	Tumlingtar	27.3400002	87.1883011	1974	27808	58095PKO	MW479763
N8	*S. progastus*	Koshi	Tumlingtar	27.3400002	87.1883011	1975	27808	58047PKO	MW479764
N8	*S. progastus*	Koshi	Tumlingtar	27.3400002	87.1883011	1976	27808	58048PKO	MW479762
N8	*S. progastus*	Koshi	Tumlingtar	27.3400002	87.1883011	1977	27808	58049PKO	MW479761
N8	*S. progastus*	Koshi	Tumlingtar	27.3400002	87.1883011	1978	27808	58050PKO	MW479760
N9	*S. richardsonii*	Koshi	Confl Num+Kotka	27.5517006	87.3016968	1964	29233	58042RKO	MW479825
N9	*S. richardsonii*	Koshi	Confl Num+Kotka	27.5517006	87.3016968	1968	29233	58046RKO	MW479823
N9	*S. richardsonii*	Koshi	Confl Num+Kotka	27.5517006	87.3016968	1969	29233	58090RKO	

S2 Table. ***Schizothorax*** **samples from Bhutan (N=19) by site and species.**

Site= sampling site (B1-N9); Species= Schizothorax sp. identification; River= basin; Location= collection area within basin (Chhu= river); Lat= Latitude; Long= Longitude; KU-T= Univ. Kansas Tissue number (KU:KUIT:#); KU-V= Univ. Kansas Voucher number (KU:KUIT:#); Haplotype= Univ. Arkansas Sequence number; Accession= GenBank Accession number. Sites depicted geographically in Fig 3.

Site	River	Location	Lat	Long	Haplotype	GenBank
B1	Wang Chhu	Nyac Chhu	27.374800	89.291000	58nyac01	
B1	Wang Chhu	Haa Chhu	27.376900	89.288800	58haac01	
B2	Punatsang Chhu	Po Chhu	27.592200	89.871600	58pots02	
B2	Punatsang Chhu	Zhawakha	27.592200	89.871600	58puza06	
B2	Punatsang Chhu	Zhawaka	27.592200	89.871600	58Ppuza01	
B3	Punatsang Chhu	Shengarong Chhu	27.526400	89.872300	58shen06	
B3	Punatsang Chhu	Toebrong Chhu	27.526400	89.872300	58toeb12	
B4	Punatsang Chhu	Dang Chhu	27.480000	89.910000	58danr04	
B4	Punatsang Chhu	Dang Chhu	27.480000	89.910000	58danr01	
B5	Punatsang Chhu	Kame Chhu	27.269500	90.036000	58kame01	
B5	Punatsang Chhu	Kami-Rong Chhu	27.269500	90.036000	58karo03	
B6	Punatsang Chhu	Tinku Chhu	27.220500	90.151900	58tink01	
B7	Punatsang Chhu	Dik Chhu	26.883000	90.271200	58dikc03	
B8	Mangde Chhu	Bert Chhu	27.145983	90.691694	58bert01	
B8	Mangde Chhu	DakPai Chhu	27.146100	90.691600	58dakp06	
B8	Mangde Chhu	DakPai Chhu	27.146100	90.691600	58dakp11	
B9	Dangem Chhu	Thungdhi Ri	27.346900	91.624390	58thun03	
B9	Dangem Chhu	Thungdhi Ri	27.346900	91.624390	58thun08	
B9	Dangme Chhu	Khardii Chhu	27.361230	91.663110	58khar01	
