# Supplementary material for: The Himalayan uplift and evolution of aquatic biodiversity across Asia: Snowtrout (Cyprininae: Schizothorax) as a test case
Source: PLoS One. 2023 Oct 24;18(10):e0289736. doi: 10.1371/journal.pone.0289736 (PMC10597529; doi:10.1371/journal.pone.0289736)
Supplement: S2 Table — Site = sampling site (B1-N9); Species = Schizothorax sp. identification; River = basin; Location = collection area within basin (Chhu = river); Lat = Latitude; Long = Longitude; KU-T = Univ. Kansas Tissue number (KU:KUIT:#); KU-V = Univ. Kansas Voucher number (KU:KUIT:#); Haplotype = Univ. Arkansas Sequence number; Accession = GenBank Accession number. Sites depicted topographically in Fig 2B. (PDF) [file pone.0289736.s003.pdf]

S2 Table. ***Schizothorax*** **samples from Bhutan (N=19) by site and species.**

Site= sampling site (B1-N9); Species= Schizothorax sp. identification; River= basin; Location= collection area within basin (Chhu= river); Lat= Latitude; Long= Longitude; KU-T= Univ. Kansas Tissue number (KU:KUIT:#); KU-V= Univ. Kansas Voucher number (KU:KUIT:#); Haplotype= Univ. Arkansas Sequence number; Accession= GenBank Accession number. Sites depicted geographically in Fig 3.

Site	River	Location	Lat	Long	Haplotype	GenBank
B1	Wang Chhu	Nyac Chhu	27.374800	89.291000	58nyac01	
B1	Wang Chhu	Haa Chhu	27.376900	89.288800	58haac01	
B2	Punatsang Chhu	Po Chhu	27.592200	89.871600	58pots02	
B2	Punatsang Chhu	Zhawakha	27.592200	89.871600	58puza06	
B2	Punatsang Chhu	Zhawaka	27.592200	89.871600	58Ppuza01	
B3	Punatsang Chhu	Shengarong Chhu	27.526400	89.872300	58shen06	
B3	Punatsang Chhu	Toebrong Chhu	27.526400	89.872300	58toeb12	
B4	Punatsang Chhu	Dang Chhu	27.480000	89.910000	58danr04	
B4	Punatsang Chhu	Dang Chhu	27.480000	89.910000	58danr01	
B5	Punatsang Chhu	Kame Chhu	27.269500	90.036000	58kame01	
B5	Punatsang Chhu	Kami-Rong Chhu	27.269500	90.036000	58karo03	
B6	Punatsang Chhu	Tinku Chhu	27.220500	90.151900	58tink01	
B7	Punatsang Chhu	Dik Chhu	26.883000	90.271200	58dikc03	
B8	Mangde Chhu	Bert Chhu	27.145983	90.691694	58bert01	
B8	Mangde Chhu	DakPai Chhu	27.146100	90.691600	58dakp06	
B8	Mangde Chhu	DakPai Chhu	27.146100	90.691600	58dakp11	
B9	Dangem Chhu	Thungdhi Ri	27.346900	91.624390	58thun03	
B9	Dangem Chhu	Thungdhi Ri	27.346900	91.624390	58thun08	
B9	Dangme Chhu	Khardii Chhu	27.361230	91.663110	58khar01	
