# Supplementary material for: The Himalayan uplift and evolution of aquatic biodiversity across Asia: Snowtrout (Cyprininae: Schizothorax) as a test case
Source: PLoS One. 2023 Oct 24;18(10):e0289736. doi: 10.1371/journal.pone.0289736 (PMC10597529; doi:10.1371/journal.pone.0289736)
Supplement: S3 Table — Species Name = Scientific name (A = Acrossocheilus; Pe = Pethia; Pu = Puntius; T = Tor; N = Neolissochilus; S = Schizothorax); Haplotype = Univ. Arkansas sequence identifier [i.e., from GenBank = letters and numbers; Univ. of Arkansas = starting with ’58’ (Univ. of Kansas tissue vouchers referenced in S1 Table)]; Location = Country, geographic region or river basin; Group = Regions; Group abbreviations are as follows: QTP = Qinghai-Tibetan Plateau; SEA = Southeastern Asia; YLTR = Yarlung-Tsangpo River; * = indicates samples that cluster with a group outside their sampling region. (PDF) [file pone.0289736.s004.pdf]

S3 Table. Samples of *Schizothorax* (N=132) and outgroup species (N=8) evaluated.

Species Name= Scientific name (A=*Acrossocheilus*; Pe=*Pethia*; Pu=*Puntius*; T=*Tor*; N=*Neolissochilus*; S=*Schizothorax*); Haplotype = Univ. Arkansas sequence identifier [i.e., from GenBank= letters and numbers; Univ. of Arkansas= beings with '58' (Univ. of Kansas tissue vouchers referenced in S1 Table)]; Location= Country, geographic region or river basin; Group= Regions as presented in Fig 1; Group abbreviations are as follows: QTP= Qinghai-Tibetan Plateau; SEA= Southeastern Asia; YLTR= Yarlung-Tsangpo River; *= indicates samples that cluster with a group outside their sampling region.

**Species Name**	**Haplotype**	**Accession #**	**Location**	**Group**
A. yunnanensis	Ayun35	KC696535	n/a	Outgroup
A. iridescens	Airi36	KC696536	n/a	Outgroup
Pu. sophore	Psup41	KF574541	n/a	Outgroup
Pe. ticto	Ptic69	AB238969	n/a	Outgroup
Pe. conchonius	Pcon51	AY004751	n/a	Outgroup
T. putitora	Tput22	JX204722	India: Beas River (Himachal Pradesh)	Outgroup
T. tor	Ttor35	KF574635	n/a	Outgroup
N. hexagonolepsis	Nhex06	NC026106	India: Jia Bhoreli River at Bhalukpong	Outgroup
S. argentatus	Sarg69	AY954269	China: Ili River (Tarim drainage)	Central Asia
S. biddulphi	Sbid66	FJ931466	China: Kezi River	Central Asia
S. eurystomus	Seur75	AY954275	China: Tashkurgan River (Tarim)	Central Asia
S. intermedius	Sint73	AY954273	China: Tashkurgan River (Tarim)	Central Asia
S. intermedius	Sint72	AY954272	China: Uqturpan	Central Asia
S. pseudoaksaiensis	Spse70	AY954270	China: Ili River (Tarim drainage)	Central Asia
S. malacanthus	Smal77	AY954277	China: Diantan (Irrawaddy drainage)	Central QTP
S. molesworthi	Smol29	DQ126129	China: Zayu (Tibet)	Central QTP
S. macropogon	Smac17	AY463517	China: Yarlung-Tsangpo River	YLTR West-A
S. waltoni_08	Swalt08	MK243412.1	China: Yarlung-Tsangpo River	YLTR West-A
S. waltoni_11	Swalt11	MK243415	China: Yarlung-Tsangpo River	YLTR West-A
S. waltoni_12	Swalt12	MK243416	China: Yarlung-Tsangpo River	YLTR West-A
S. waltoni_13	Swalt13	MK243417	China: Yarlung-Tsangpo River	YLTR West-A

S. waltoni_14	Swalt14	MK243418	China: Yarlung-Tsangpo River	YLTR West-A
S. waltoni_15	Swalt15	MK243419	China: Yarlung-Tsangpo River	YLTR West-A
S. waltoni_16	Swalt16	MK243420	China: Yarlung-Tsangpo River	YLTR West-A
S. waltoni_17	Swalt17	MK243421	China: Yarlung-Tsangpo River	YLTR West-A
S. wangchiachii	Swan81	HQ198881	China: Yarlung-Tsangpo River	YLTR West-B
S. oconnori	Socon10	KT188623	China: Yarlung-Tsangpo River	YLTR West-B
S. oconnori	Socon24	KT188637	China: Yarlung-Tsangpo River	YLTR West-B
S. oconnori	Socon28	KT188641	China: Yarlung-Tsangpo River	YLTR West-B
S. oconnori	Socon50	KT188663	China: Yarlung-Tsangpo River	YLTR West-B
S. species	58haac01		Bhutan: Haa Chhu (Wang Chhu)	Bhutan-1A
S. species	58nyac01		Bhutan: Wang Chhu (Nya Chhu)	Bhutan-1A
S. species	58dakp06		Bhutan: Dakpai Chhu (Mangde Chhu)	Bhutan-1B
S. species	58dakp11		Bhutan: Dakpai Chhu (Mangde Chhu)	Bhutan-1B
S. species	58thun03		Bhutan: Shengarong Chhu (Punatsang Chhu)	Bhutan-1B
S. oconneri	Socon58	KT188671	China: Yarlung-Tsangpo River	YLTR East
S. oconneri	Socon59	KT188672	China: Yarlung-Tsangpo River	YLTR East
S. oconneri	Socon60	NC020781	China: Yarlung-Tsangpo River	YLTR East
S. species	58bert01		Bhutan: Berti Chhu (Mangde Chhu)	Bhutan-1C
S. species	58danr04		Bhutan: Dang Chhu (Punatsang Chhu)	Bhutan-1C
S. species	58khar01		Bhutan: Khardii Chhu (Dangme Chhu)	Bhutan-1C
S. species	58pots02		Bhutan: Po Chhu (Punatsang Chhu)	Bhutan-1C
S. species	58puza01		Bhutan: Zhawaka Chhu (Punatsang Chhu)	Bhutan-1C
S. species	58thun08		Bhutan: Shengarong Chhu (Punatsang Chhu)	Bhutan-1C
S. chongi	Scho18	DQ126118	China: Jiulong River (Yangtze drainage)	E-QTP/ SEA-B
S. davidi	Sdav13	DQ126113	China: Ya'an (Yangtze drainage)	E-QTP/ SEA-B
S. dolichonema	Sdol17	DQ126117	China: Jiulong River (Yangtze drainage)	E-QTP/ SEA-B
S. dulongensis	Sdul84	AY954284	China: Tengchong (Irrawaddy drainage)	E-QTP/ SEA-A
S. gongshanensis	Sgon79	AY954279	China: Fugong (Salween drainage)	E-QTP/ SEA-C
S. gongshanensis	Sgon80	AY954280	China: Fugong (Salween drainage)	E-QTP/ SEA-C

S. griseus	Sgri53	AY954253	China: Yongping (Mekong draiange)	E-QTP/ SEA-C
S. kozlovi	Skoz12	DQ126112	China: Jiulong River (Yangtze drainage)	E-QTP/ SEA-B
S. lantsangensis	Slan82	DQ646882	China: Lancang River (Mekong drainage)	E-QTP/ SEA-C
S. lissolabiatus	Slis59	KP796159	China: Nujiang River (Salween drainage)	E-QTP/ SEA-C
S. lissolabiatus	Slis67	KP796167	China: Nujiang River (Salween drainage)	E-QTP/ SEA-C
S. meridionalis	Smer87	AY954287	China: Yingjiang (Irrawaddy drainage)	E-QTP/ SEA-A
S. nukiangensis	Snuk25	DQ126125	China: Chalong (Tibet) (Salween drainage)	E-QTP/ SEA-C
S. prenanti	Spre62	AY954262	China: Fenjie (Yangtze drainage)	E-QTP/ SEA-B
S. prenanti	Spre61	AY954261	China: Judian (Yangtze drainage)	E-QTP/ SEA-B
S. rotundimaxillaris	Srot83	AY954283	China: Tengchong (Irrawaddy drainage)	E-QTP/ SEA-A
S. wangchiachii	Swan21	DQ126121	China: Jiulong (Yangtze drainage)	E-QTP/ SEA-B
S. yunnanensis	Syun71	KP796171	China: Nujiang River (Salween drainage)	E-QTP/ SEA-A
S. yunnanensis	Syun69	KP796169	China: Nujiang River (Salween drainage)	E-QTP/ SEA-A
S. yunnanensis	Syun86	AY954286	China: Yingjiang (Irrawaddy drainage)	E-QTP/ SEA-A
S. yunnanensis	Syun52	AY954252	China: Yongping (Mekong drainage)	E-QTP/ SEA-C
S. species	58danr01		Bhutan: Dang Chhu (Punatsang Chhu)	Bhutan-2
S. species	58dikc03		Bhutan: Dik Chhu (Punatsang Chhu)	Bhutan-2
S. species	58kame01		Bhutan: Kame Chhu (Punatsang Chhu)	Bhutan-2
S. species	58karo03		Bhutan: Kami-Rong Chhu (Punatsang Chhu)	Bhutan-2
S. species	58puza06		Bhutan: Zhawaka Chhu (Punatsang Chhu)	Bhutan-2
S. species	58shen06		Bhutan: Shengarong Chhu (Punatsang Chhu)	Bhutan-2
S. species	58tink01		Bhutan: Tinku Chhu (Punatsang Chhu)	Bhutan-2
S. species	58toeb12		Bhutan: Toebrong Chhu (Punatsang Chhu)	Bhutan-2
S. esocinus	Seso50	KP712250	Nepal: Melamchi River	Koshi River
S. progastus	58047PKO	MW479764	Nepal: Tumlingtar Region	Koshi River
S. progastus	58048PKO	MW479762	Nepal: Tumlingtar Region	Koshi River
S. progastus	58049PKO	MW479761	Nepal: Tumlingtar Region	Koshi River
S. progastus	58050PKO	MW479760	Nepal: Tumlingtar Region	Koshi River
S. progastus	58095PKO	MW479763	Nepal: Tumlingtar Region	Koshi River
S. richardsonii	Sric49	KP712249	Nepal: Indrawati River	Koshi River

S. richardsonii	58042RKO	MW479825	Nepal: Arun River	Koshi River
S. richardsonii	58046RKO	MW479823	Nepal: Arun River	Koshi River
S. richardsonii	58090RKO		Nepal: Arun River	Koshi River
S. progastus	58038PGA	MW479775	Nepal: Kali Gandaki River	Gandaki River
S. progastus	58039PGA	MW479774	Nepal: Kali Gandaki River	Gandaki River
S. progastus	58040PGA	MW479776	Nepal: Kali Gandaki River	Gandaki River
S. progastus	58052PGA	MW479771	Nepal: Rahughat River	Gandaki River
S. progastus	58072PGA	MW479770	Nepal: Rahughat River	Gandaki River
S. progastus	58075PGA	MW479769	Nepal: Rahughat River	Gandaki River
S. progastus	58076PGA	MW479768	Nepal: Rahughat River	Gandaki River
S. progastus	58077PGA	MW479767	Nepal: Rahughat River	Gandaki River
S. progastus	58073PGA		Nepal: Kali Gandaki River	Gandaki River
S. richardsonii	58074RGA	MW479799	Nepal: Kali Gandaki River	Gandaki River
S. richardsonii	58085RGA	MW479798	Nepal: Kali Gandaki River	Gandaki River
S. richardsonii	58088RGA	MW479796	Nepal: Kali Gandaki River	Gandaki River
S. richardsonii	58096RGA	MW479794	Nepal: Kali Gandaki River	Gandaki River
S. richardsonii	58102RGA	MW479791	Nepal: Kali Gandaki River	Gandaki River
S. richardsonii	58106RGA	MW479788	Nepal: Kali Gandaki River	Gandaki River
S. richardsonii	58113RGA	MW479786	Nepal: Kali Gandaki River	Gandaki River
S. richardsonii	58114RGA	MW479785	Nepal: Kali Gandaki River	Gandaki River
S. richardsonii	58115RGA	MW479784	Nepal: Kali Gandaki River	Gandaki River
S. richardsonii	58117RGA	MW479783	Nepal: Kali Gandaki River	Gandaki River
S. richardsonii	58120RGA	MW479780	Nepal: Kali Gandaki River	Gandaki River
S. progastus	58122PKA		Nepal: Jhugala	Karnali River
S. progastus	58123PKA		Nepal: Jhugala	Karnali River
S. richardsonii	58020RKA	MW479810	Nepal: Srikot	Karnali River
S. richardsonii	58021RKA	MW479809	Nepal: Srikot	Karnali River
S. richardsonii	58036RKA	MW479818	Nepal: Srikot	Karnali River
S. richardsonii	58053RKA	MW479808	Nepal: Gumgarh River (Bhotechaur)	Karnali River
S. richardsonii	58078RKA	MW479807	Nepal: Srikot	Karnali River

S. richardsonii	58079RKA	MW479806	Nepal: Srikot	Karnali River
S. richardsonii	58082RKA	MW479778	Nepal: Jhugala	Karnali River
S. richardsonii	58083RKA	MW479804	Nepal: Jhugala	Karnali River
S. richardsonii	58084RKA	MW479803	Nepal: Jhugala	Karnali River
S. richardsonii	58109RKA	MW479787	Nepal: Gumgarh River (Bhotechaur)	Karnali River
S. richardsonii	58110RKA	MW479802	Nepal: Gumgarh River (Bhotechaur)	Karnali River
S. richardsonii	58112RKA	MW479800	Nepal: Gumgarh River (Bhotechaur)	Karnali River
S. macrophthalmus	58019MR	MW479837	Nepal: Rara Lake	Karnali River
S. macrophthalmus	58031MR	MW479836	Nepal: Rara Lake	Karnali River
S. macrophthalmus	58061MR	MW479833	Nepal: Rara Lake	Karnali River
S. nepalensis	58002NR	MW479846	Nepal: Rara Lake	Karnali River
S. nepalensis	58003NR	MW479814	Nepal: Rara Lake	Karnali River
S. nepalensis	58011NR	MW479842	Nepal: Rara Lake	Karnali River
S. nepalensis	58033NR		Nepal: Rara Lake	Karnali River
S. nepalensis	Snep07	AP011207	Nepal: Rara Lake	Karnali River
S. raraensis	58054RR	MW479855	Nepal: Rara Lake	Karnali River
S. raraensis	58056RR	MW479854	Nepal: Rara Lake	Karnali River
S. raraensis	58057RR	MW479853	Nepal: Rara Lake	Karnali River
S. esocinus	Seso82	KT210882	Pakistan: Northern	Ganges River*
S. plagiostomus	SplaP1	KR232369	Pakistan: Neelum/Jhelum R.	Ganges River*
S. progastus	58126PKA		Nepal: Jhugala (Karnali R.)	Ganges River*
S. richardsonii	Sric01	JX485901	India: Upper Ganges	Ganges River
S. richardsonii	Sric02	JX485902	India: Upper Ganges	Ganges River
S. richardsonii	Sric82	JX485882	India: Upper Ganges	Ganges River
S. richardsonii	Sric83	JX485883	India: Upper Ganges	Ganges River
S. richardsonii	Sric84	JX485884	India: Upper Ganges	Ganges River
S. plagiostomus	SplaP2	KT184924	Pakistan: Northern	Indus River
S. progastus	SproP1	KX254914	Pakistan: Neelum/ Jhelum R.	Indus River
S. progastus	SproP2	KX254915	Pakistan: Neelum/ Jhelum R.	Indus River
S. richardsonii	SricKO	KC790369	India: Ratighat (Koshi Tributary)	Indus River*
