# Supplementary figures and images for: The Himalayan uplift and evolution of aquatic biodiversity across Asia: Snowtrout (Cyprininae: Schizothorax) as a test case
Source: PLoS One. 2023 Oct 24;18(10):e0289736. doi: 10.1371/journal.pone.0289736 (PMC10597529; doi:10.1371/journal.pone.0289736)

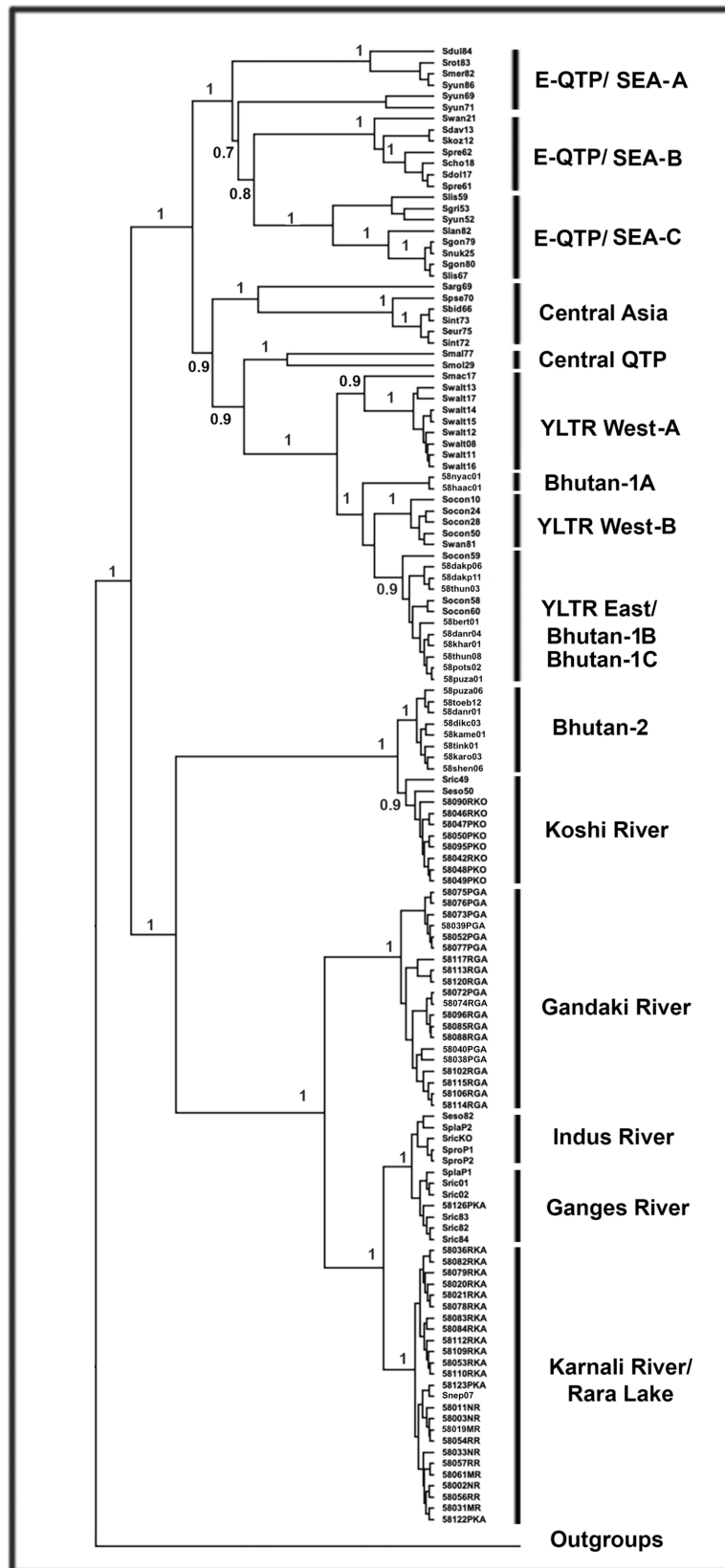

Supplement: S1 Fig — Phylogenetic relationships among snowtrout (Schizothorax; Cyprinidae) and outgroup taxa derived from sequence analysis of the cytochrome-b mitochondrial gene (1,140 bp, 140 haplotypes). Individuals are coalesced into geographic regions, with abbreviations defined as: E-QTP/ SEA = Eastern Qinghai-Tibetan Plateau/ Southeast Asia, with three subclades indicated by A, B, and C; YLTR = Yarlung-Tsangpo River, with subclades indicated by West-A, West-B, and East. Numbers at nodes represent Bayesian posterior probabilities. Sample metadata in S3 Table. (PDF) [file pone.0289736.s005.pdf]
